# A novel FIKK kinase regulates the development of mosquito and liver stages of the malaria

**DOI:** 10.1038/srep39285

**Published:** 2016-12-20

**Authors:** Dabbu Kumar Jaijyan, Praveen Kumar Verma, Agam Prasad Singh

**Affiliations:** 1 Infectious Diseases Laboratory, National Institute of Immunology, Aruna Asaf Ali Marg, New Delhi-110067, India; 2 Plant Immunity Laboratory, National Institute of Plant Genome Research, Aruna Asaf Ali Marg, New Delhi-110067, India

## Abstract

Protein phosphorylation is the most important post-translational event in the regulation of various essential signaling pathways in a cell. Here, we show the functional characterization of a FIKK family protein kinase of the rodent malaria parasite (*Pb*MLFK), which is expressed only in mosquito and liver stages and contains two functional C-terminal PEXEL motifs. We demonstrate that this protein plays a role in mosquito and liver stages of parasite growth. The oocysts of *Pb*MLFK-deficient parasites produced 4-fold fewer sporozoites. In the liver of infected mice, *Pb*MLFK-deficient parasites grew 100-fold less than did wild type parasites. We also show that the C-terminal domain of this protein has a functional serine-threonine kinase and that its activity was inhibited by a known PKA inhibitor. Transcriptome analysis of infected host cells suggests that in absence of this protein expression of the 288 host mRNAs are perturbed which are primarily associated with the immune system, cell cycle and metabolism.

Malaria is a devastating infectious disease that causes ~200 million clinical cases and ~600,000 deaths (mainly children) each year^1^. The life cycle of the malaria parasite is complex and is completed in two different hosts: mosquitoes and humans (pre-erythrocytic and erythrocytic schizogony)^2^. Once the parasite has invaded a host cell, it hides itself inside the parasitophrous vacuole (PV). The PV physically separates the parasite from host cytosol, and further development of the parasite occurs within PV^3^. Host cell invasion and successful parasite development inside the cell depend crucially on the export of parasite proteins to the host cell cytosol. The conserved *Plasmodium* export element (PEXEL) mediates the transport of parasite proteins across the PV membrane (PVM) in both the blood stage^4^,^5^ and the liver stage of the parasite^6^. Parasite proteins are also exported across PVM via PEXEL independent pathway and such proteins are termed as pexel negative exported proteins (PNEP)^7^. Growing parasites face diverse cellular environments within the different host tissues. To overcome hurdles in the path to productive infection, parasites have evolved (a) effective and adaptive signaling mechanisms to respond to specific environments and (b) ways to protect themselves from host immune responses. Similar to other higher organisms, malaria parasite kinases regulate various essential biological processes such as the cell cycle, cell-to-cell signaling, morphogenesis, gene expression, cell proliferation and differentiation^8^. Previous studies have demonstrated that kinases have many functions throughout the *Plasmodium* life cycle^9^,^10^,^11^,^12^,^13^,^14^,^15^,^16^,^17^. In the case of *Plasmodium falciparum*, 85 putative eukaryotic-like protein kinases (ePKs) have been reported^18^, although only a few of them have been characterized. Among them, a novel putative kinase family called FIKK (phenylalanine, isoleucine, lysine, lysine) was identified^19^. In *P. falciparum*, 18 of the 20 FIKK kinase genes are proposed to encode functional kinases, 16 of which are likely to be exported to the infected host cell^20^. Some of these genes have been experimentally tested for their ability to export to the host cell and for their kinase activity. Most of the FIKK proteins are genomically located in subtelomeric regions of chromosomes^20^,^21^. Kinases regulate various essential processes related to parasite development, infectivity and host modulation; therefore, kinases are fascinating drug targets. Previously, a study^22^ showed that a bumped kinase inhibitor blocked parasite transmission in mosquitoes via the inhibition of calcium dependent protein kinase 4 (CDPK4).

The genome of the rodent malaria parasite, *P. berghei* ANKA (PbA) has a single FIKK family kinase encoded by the PBANKA_1225000 gene (PbA_1225000). In this study, we demonstrated a role for this kinase. A previous study by Tewari *et al*.^23^ concluded that this gene is likely essential and refractory to disruption. As shown below, the PbA_1225000 coded protein is specifically expressed in mosquito and liver stages of malaria parasites; therefore, we named it *Pb*MLFK (*P. berghei* Mosquito and Liver stage specific FIKK Kinase). Because *Pb*MLFK protein does not express in blood stage parasites [although transcript was detected in blood stage schizonts^24^], one is able to delete this gene in the blood stage without affecting parasite growth/development. We found that *PbMLFK* gene knockout leads to a 100-fold reduction in liver stage parasite burden, thus showing stage-specific function. We also showed that *Pb*MLFK has a serine-threonine kinase activity and that it is expressed from the early oocyst stage (day 4) in mosquitos to the late liver stage in mammalian hosts.

## Results

### *PbMLFK* encodes a conserved serine-threonine (S/T) kinase

**Figure 1:**
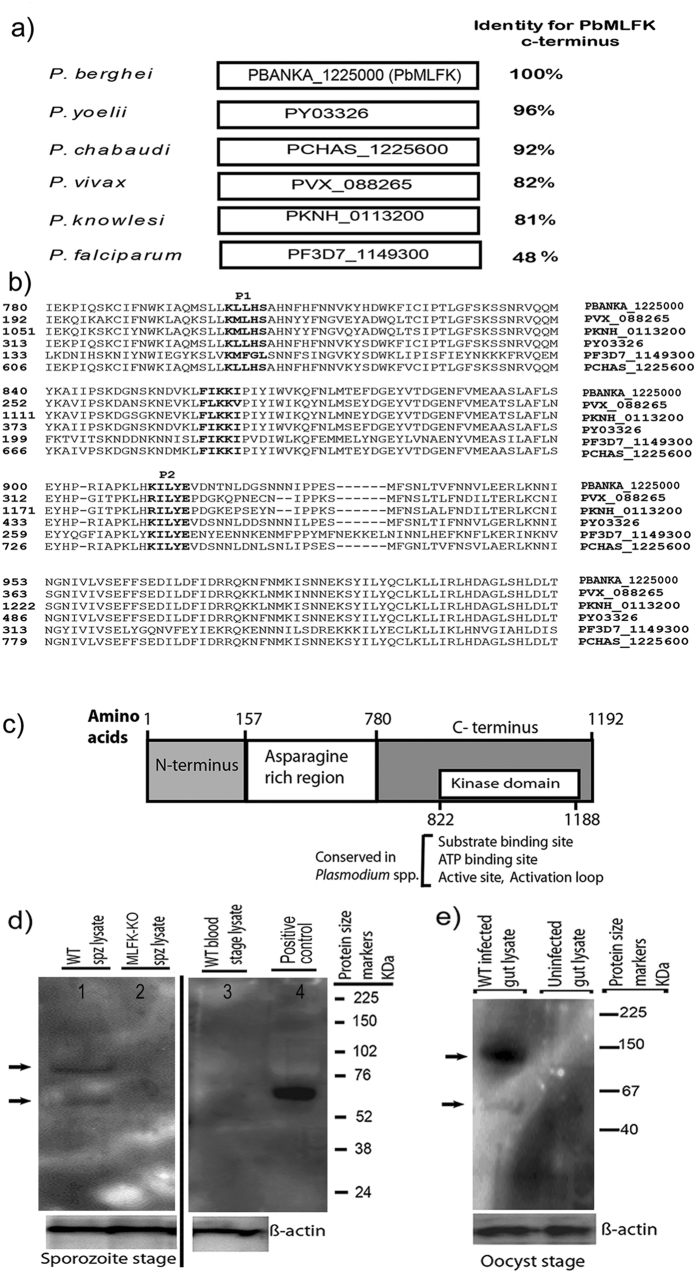
*PbMLFK* encodes a conserved serine-threonine kinase protein. (**a**) Sequence identity of the C-terminus of *Pb*MLFK compared with the orthologous proteins in other *Plasmodium* species. (**b**) Multiple sequence alignment (MSA) of *Pb*MLFK with orthologous proteins from other *Plasmodium* species. The GenBank accession numbers for the *Plasmodium* proteins are indicated next to the protein sequence. Conserved PEXEL motifs and FIKK sequences are shown in bold letters. (**c**) Graphical representation of different domains present in *Pb*MLFK, showing the conserved C-terminal region containing the kinase domain (a.a. 699–1192). (**d**) Anti-*Pb*MLFK peptide mouse polyclonal antibody detects *Pb*MLFK in WT sporozoite lysate (arrow marks) but not in *PbMLFK*-KO sporozoite lysates or *Pb*A blood stage lysate. Lane1, *Pb*A sporozoite (5 × 10e^6^) lysate; Lane 2, *PbMLFK*-KO (described later, refer to Fig. 4) sporozoite (5 × 10e^6^) lysate; Lane 3, *Pb*A blood stage (5 × 10e^6^) lysate; Lane 4, positive control (*E. coli* recombinant and partially purified C-terminal PbMLFK), Right side to lane 4-protein size markers. (**e**) Western blot analysis of full-length *Pb*MLFK: IP lysate from 150 infected mosquito midguts (left side lane, oocyst) and 150 uninfected mosquito midgut IP lysate (right side lane, control). (**d**,**e**) both show a full length protein (~140 kDa, arrow mark) and smaller fragment (58 ± 2 kDa, arrow mark). For (**d**,**e**) full blots are provided at the end of supplementary file.

*Pb*MLFK [PBANKA_1225000] is a protein kinase member of the FIKK family and contains a conserved putative PEXEL motif and a FIKK sequence at its c-terminus. Figure 1a shows the percent identity of the *Pb*MLFK C-terminal region compared with orthologous proteins in other species of *Plasmodium*. PEXEL motifs (predicted) in *Pb*MLFK are shown in bold letters in Fig. 1b. Bioinformatics analysis predicted that the C-terminal region of *Pb*MLFK consists of an ATP binding site, substrate-binding site and a kinase domain (Fig. 1c). Among all *P. falciparum* FIKK kinases, *Pf*-FIKK8 [PF3D7_0805700] showed the highest amino acid sequence homology (score = 1965 and PN value of 5.9e-214) and was syntenic to *Pb*MLFK. The C-terminus of *Pb*MLFK (amino acids 699 to 1192) showed significant homology to all *Pf*-FIKK kinases, which indicated that it could be a conserved functional domain.

### *Pb*MLFK is expressed during mosquito and liver stages of malaria parasite

A previous report^4^ documented that another FIKK member PF3D7_1149300 is transcribed in the sporozoite stage and contains a putative PEXEL motif hence to explore the role played by sporozoite stage-specific S/T kinases, we characterized *Pb*MLFK biochemically and by genetic modification. To investigate the expression of *Pb*MLFK during the malaria parasite life cycle, we generated mouse polyclonal sera against peptides (YETNNDNHSN and KCSDPLQED) derived from C-terminus of *Pb*MLFK and which were conjugated with KLH (Keyhole limpet hemocyanin). Western blot analysis using the anti-*Pb*MLFK peptide antibody, showed that the sera specifically recognized *Pb*MLFK in wild type (WT) sporozoites (Fig. 1d) and oocyst lysates (Fig. 1e) but not in *PbMLFK* knockout (*PbMLFK* -KO) sporozoites or wild type blood stage lysates (Fig. 1d). Furthermore, western blot analysis of lysates from the sporozoites also showed that *Pb*MLFK is an ~140 kDa protein (Fig. 1d, top arrow). Figure also shows that *Pb*MLFK is possibly processed either proteolytically or spliced (Lower arrow mark in Fig. 1d and e) to give a smaller domain (around 55 to 60 kDa) which is simlar in size of C-terminal portion of the protein [calculated mass of *Pb*MLFK C-terminal fragment (amino acids 699–1192) is 54.34 kDa].

**Figure 2:**
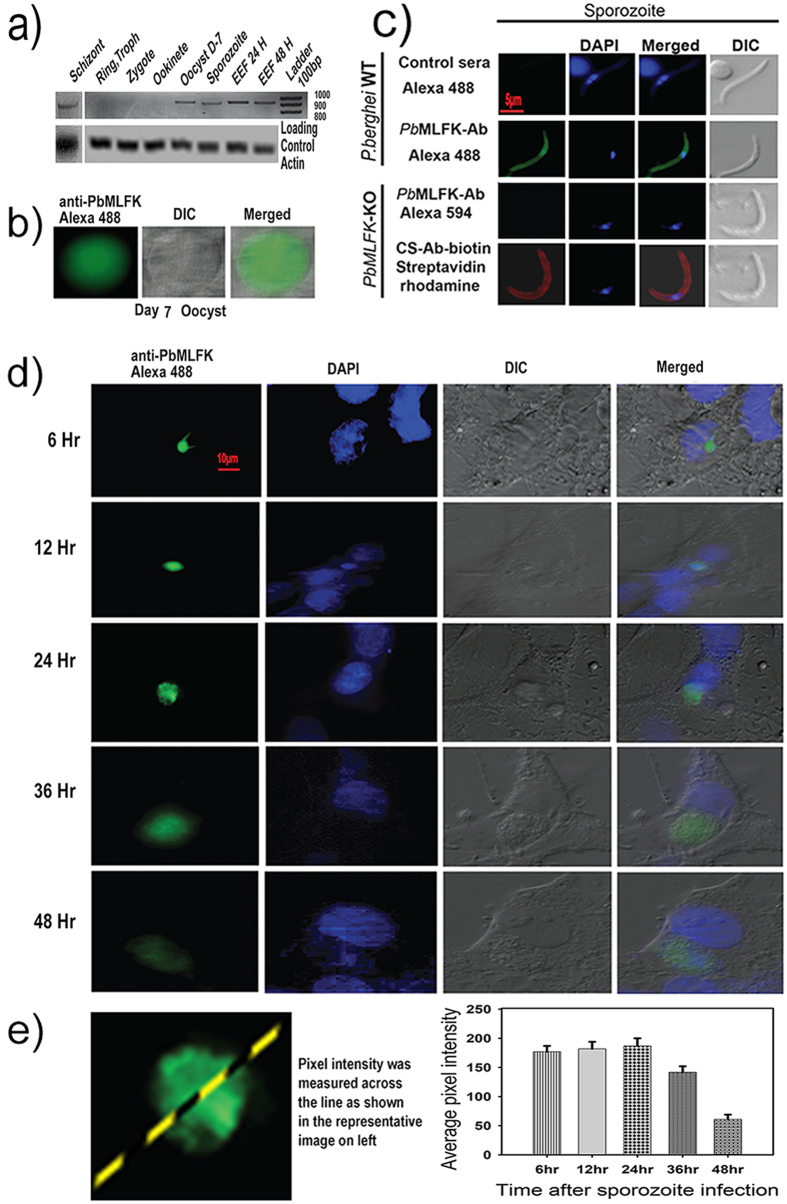
*Pb*MLFK expresses in oocyst, sporozoite and liver stage of *Pb*A parasites. (**a**) *Pb*MLFK expression during various life stages of the *Plasmodium*. RNA isolated from various stages, as denoted in figure, was converted into cDNA which was then used as template in a PCR. A portion of *Pb*MLFK was amplified using specific primers. Expected size PCR product was observed and figure shows transcripts in Schizont, Oocyst, Sporozoites and EEFs (liver stages). *Pb*MLFK does not show detectable transcripts in Ring, Trophozoite, Zygote and ookinete of *Plasmodium.* Full image is provided at the end of supplementary file (**b**) Fluorescence image (green) showing *Pb*MLFK expression during the oocyst stage. The differential interference contrast (DIC) image shows the morphology of oocysts in the midgut of infected mosquitoes. (**c**) Fluorescence image (green) showing *Pb*MLFK expression and localization in a *Pb*A sporozoite. The sporozoite nucleus is visualized by staining with DAPI (blue), and sporozoite morphology is represented by the DIC image. The merged image represents the overlay of all three images. (**d**) IFA results showing *Pb*MLFK expression during the liver stage of *Pb*A. *Pb*MLFK is expressed from early through late liver stage. (**e**) Average pixel intensity across the line passing through the middle of the parasite (EEF). Fluorescence intensity decreases by a third at late time points (approximately 48 hr) compared with the intensity observed at 24 hr.

To find out the *Pb*MLFK transcript profile, we isolated RNA from various life stages of the parasite and after its conversion into complementory DNA (cDNA) used it as template in a polymerase chain reaction (PCR) to test the presence of RNA. We found *Pb*MLFK transcripts in blood stage Schizont, Oocyst, sporozoite and liver stages (also termed as exo-erythrocytic forms or EEF) but not in the Ring, Trophozoite, zygote and ookinetes (Fig. 2a). Further, to find out protein expression when analyzing its expression in oocysts via immunofluorescence assay (IFA), we found that *Pb*MLFK expressed in the oocyst stage (Fig. 2b). In salivary gland sporozoites IFA results revealed that *Pb*MLFK was present in the cytosol (Fig. 2c). IFA also showed protein expression from the early (6 hr) to late liver stage (48 hr) in HepG2 cells (Fig. 2d). The graph in Fig. 2e shows an average pixel intensity at indicated time points of an EEF growth. Finally, IFA on thin blood smears (containing Ring, Trophozoite, scizont and gamete stages) (Fig. S1A) showed that there was no expression during the blood stage. Therefore, we concluded that *Pb*MLFK Protein expression starts in the oocyst stage of the malaria parasite, is maintained within sporozoites and during the liver stage and is paused during the late liver stage.

### *Pb*MLFK contains two functional PEXEL motifs in the C-terminal domain

**Figure 3:**
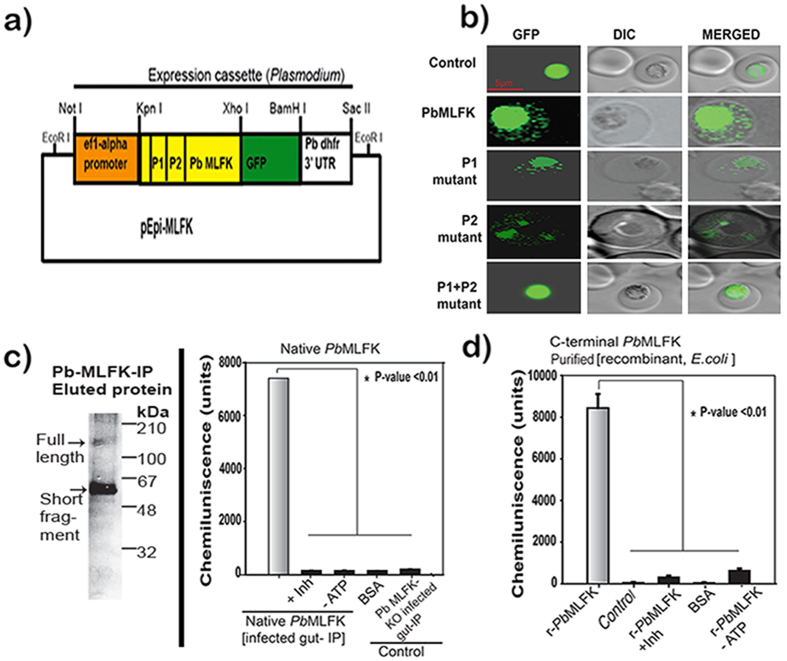
(**a**) Graphical representation of the episomal vector (Epi-*PbMLFK*) that was used for the PEXEL motif functional assay. The *Pb*MLFK C-terminal region was cloned at the *Kpn*I and *Xho*I sites in the episomal vector. Vector consists of an EF1α promoter that drives the expression of the *PbMLFK* gene, all fused in frame with *GFP* at the C-terminus which is followed by Pbdhfr 3′ UTR (**b**) Fluorescence image of the Epi-*PbMLFK*-WT transfected parasite showing the export of *Pb*MLFK into the cytosol of infected RBC (2^nd^ row). The parasite transfected with the Epi-*PbMLFK* PEXEL single mutant (P1) construct (3^rd^ row) and single mutant (P2) construct (4^th^ row) shows the export of *Pb*MLFK into the cytosol of infected RBCs, although at a reduced level compared with the WT motif. The parasite transfected with the Epi-*PbMLFK* PEXEL double (P1and P2) mutant construct (5^th^ row) did not show the export of *Pb*MLFK into the cytosol of infected RBCs, and the green signals are restricted to the confines of the parasite similar to the GFP control (1^st^ row). (**c**) Kinase activity assay with the immunoprecipitated (IP) protein from the lysate. Lysate was extracted from the 150 infected mosquito midguts using the anti-*Pb*MLFK peptide antibody. IP-elutes were used in assays performed with a phospho-serine/threonine antibody-based ELISA kit. Full image is provided at the end of supplementary file (**d**) Kinase activity assay using C-terminal *Pb*MLFK (500 ng per assay) produced in *E. coli* and affinity purified using glutathione S-transferase (GST) beads. Control represents proteins purified using empty vector transformed *E. coli* and using identical conditions for expression, purification.

*Pb*MLFK contains two PEXEL motifs, KLLHS (P1) and KILYE (P2), which are shown in bold letters in Fig. 1b, and depicted as P1 and P2, respectively, in Fig. 3a. Multiple alignment of amino acid sequences showed that both motifs are conserved throughout the *Plasmodium* species. In the alignment, the FIKK motif is shown in bold letters in Fig. 1b. *Pb*MLFK does not contain an obvious signal peptide at its N-terminus; instead, it contains a transmembrane (TM) domain in its C-terminus (Fig. S2) that can act as a translocation signal to traffic into the endoplasmic reticulum. The *Pb*MLFK TM domain likely drives its export to host cells, as previously reported for PfEMP1, which also contain a PEXEL motif and a TM domain but no signal sequence^25^. To assay PEXEL-mediated export, which is feasible only in blood stage parasites and done with the help of an episomal vector^4^,^5^,^26^, we tested pexel motif of PbMLFK wherein we used a modified vector, as shown in Fig. 3a. The construct contains the wild type *Pb*MLFK C-terminal sequence in frame with a C-terminal GFP. We observed *Pb*MLFK-GFP in the cytosol of parasitized RBC (Fig. 3b, 2^nd^ row). Using the above functional assay, we found that both motifs were active. By mutating PEXEL motifs, we found that the export of *Pb*MLFK was completely aborted only when both motifs were mutated simultaneously (Fig. 3b, bottom row). Although we observed export in the blood stage pexel motif assay, in the hepatocytes infected with liver stage parasites; we could not confirm the export, by indirect Immunoflurescence assay. Currently transfection/electroporation of the liver stage parasites method is not available hence the pexel motif assay as described above can not be performed in infected hepatocytes.

### *Pb*MLFK is a functional kinase

*Pb*MLFK contains a conserved serine-threonine (S/T) kinase domain in its C-terminus; therefore, we tested the kinase activity. We used full-length *Pb*MLFK obtained from immunoprecipitations (IP) from the midgut of infected mosquitoes using anti-*Pb*MLFK antibody. The results showed that the IP lysate containing the full-length *Pb*MLFK had kinase activity compared with controls (Fig. 3c). We also evaluated the kinase activity with C-terminal domain recombinant protein [GST-column purified], and the results confirmed that it had comparable kinase activity (Fig. 3d). Taken together, these findings show that *Pb*MLFK is a functional S/T kinase and that the kinase activity is present in its C-terminal region. *Pb*MLFK ortholog in *P. falciparum (Pf*-FIKK8) was recently shown to have active kinase domain in its C-terminus which constitute a core stable domain and is conserved in all FIKK proteins^27^, this finding also lends support to our findings. *Pf*-FIKK8 which is 1457 amino acid long could be expressed only when a minimal domain ranging from 409 to 499 amino acids in length from C-terminus (core domain, 959–1457 biggest and 1049–1457 smallest) was used for expression. A bigger size or smaller than core domain did not express and it was also true for related Cryptosporidium [cgd5_4390] FIKK kinase [supplementary data in ref. 27]. Using a protein kinase-A inhibitor (triciribine), we were able to completely block the kinase activity of *Pb*MLFK (Fig. 3c and d).

### *PbMLFK*-KO parasites are defective in the oocyst stage

**Figure 4:**
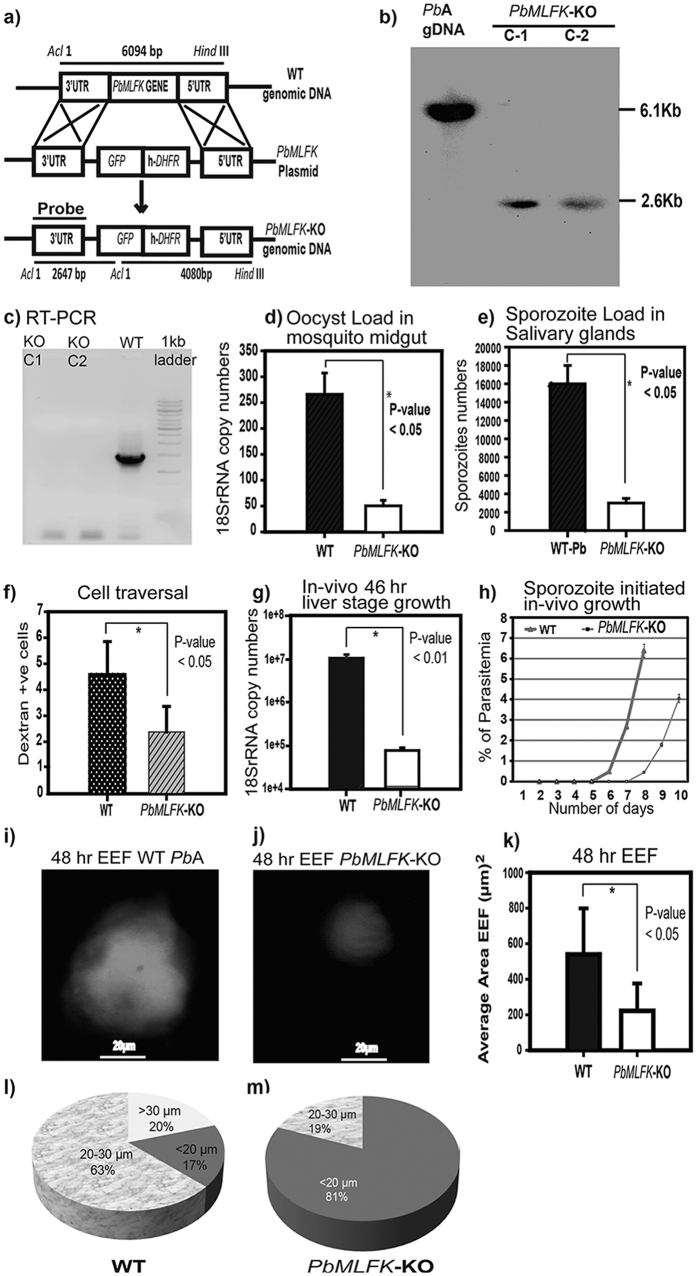
(**a**) Replacement strategy for the *PbMLFK* gene knockout in the *Pb*A parasite. Figure shows a graphical representation of the targeted gene disruption strategy for the *PbMLFK* gene knockout parasite line. Double homologous recombination between the targeting construct and the *PbMLFK* genomic locus generates a parasite line lacking the *PbMLFK* gene. Restriction sites are also shown for WT and *PbMLFK*-KO genomic DNA (gDNA). (**b**) Southern blot hybridization to confirm the disruption of the *PbMLFK* gene. The WT and *PbMLFK*-KO gDNA was digested with *Hind*III and *Acl*I. 3′UTR probe detects a band of 6 kb for WT and 2.6 kb for *PbMLFK-*KO digested gDNA, as described in (**a**). Full blot is provided at the end of supplementary file (**c**) Complementary DNA (cDNA) was made with RNA isolated from *PbMLFK*-KO sporozoites or *Pb*A wild type sporozoites and PCR analysis was performed on cDNA using the primers specific for *PbMLFK* gene. Wild type sporozoite lane show an expected size band while in knockout sporozoite lanes there was no PCR product. (**d**) WT and *PbMLFK*-KO oocyst parasite load in the midgut of infected mosquitoes as analyzed by real-time PCR using the 18 S rRNA primers and gDNA from infected mosquito midguts as a template. (**e**) WT and *PbMLFK* KO parasite sporozoite numbers in the salivary glands of infected mosquitoes. (**f**) Cell traversal assay for WT and *PbMLFK-*KO. (**G**) *In vivo* growth assay: Parasite load was checked 46 hpi in the livers of *PbMLFK*-KO or WT sporozoite-infected mice by measuring the parasite 18 S rRNA transcript copy numbers. Each bar shows the average value of parasite 18 S rRNA from the five infected mice. Error bars represent the standard deviation of each group. (**h**) BALB/c mice were intravenously injected with 6000 WT or *PbMLFK*-KO sporozoites, and parasitemia was monitored each day by Giemsa-stained thin blood smears from the infected mice. Each data point shows an average parasitemia of five mice. (**i**,**j**) Fluorescence images of the *in-vitro* 48 hour grown *Pb*A parasites (**i**) and *PbMLFK*-KO parasites (**j**). (**k**) Average area calculated from the images of liver stage parasites within HepG2 cells infected with *PbMLFK*-KO or WT parasites. Each bar represents the average value from 50 parasites. (**l**,**m**) Pie chart showing WT (**l**) or *PbMLFK* KO (**m**) liver stage parasite population distributions in different size groups based on parasite diameter.

To gain a better understanding of the function of *Pb*MLFK during the life cycle of *Pb*A, we generated a parasite line deficient in the *PbMLFK* gene (gene knockout) using the double homologous recombination method (Fig. 4a). In a previous study, Tewari *et al*.^23^ concluded that this gene is likely essential and refractory to disruption. Because *Pb*MLFK protein was not detected in the blood stage parasite (this study), one should be able to delete *PbMLFK* from the blood stage without affecting parasite growth and development. To achieve this, a targeting construct was prepared that contained the 5′ untranslated terminal region (UTR) and 3′UTR of the *PbMLFK* gene (no part of the gene was included in the construct) flanking human dihydrofolate reductase (h*DHFR)* and green flourescent protein (*GFP)* cassettes. The linearized construct (*Sca*I-digested) designed for knocking out the gene was transfected into purified schizonts, and subsequently clonal populations of transgenic parasites (knockouts) were obtained. Southern blot hybridization and reverse transcription PCR (RT-PCR) confirmed gene deletion in the *PbMLFK*-KO parasite line (Fig. 4b and c). Expected band sizes of 2.6 kb and 6.1 kb were detected using a 3′UTR probe with *PbMLFK*-KO or WT genomic DNA, respectively, that was digested with *Acl*I and *Hind*III restriction enzymes. This expected outcome is also explained in the knockout strategy in Fig. 4a. PCR performed (using the gene specific primers) on cDNA made from *PbMLFK*-KO sporozoites RNA further confirmed the loss of *PbMLFK*.

Next, to analyze the phenotype of *PbMLFK*-KO, we checked the parasite loads (oocyst) in the mosquito midgut and observed that there was 4-fold less load compared with *Pb*A-WT parasites (Fig. 4d). The defect in oocyst was not due to reduced size but it was because of reduced number. In wild type we found 60 ± 10 while in *PbMLFK*-KO it was 15 ± 5 oocyst per midgut (average of 30 midguts, n = 3). We also observed 50% less sporozoites per oocyst in case of *PbMLFK*-KO compared to wild type (average of 50 midguts, n = 2). We then determined the number of sporozoites in the salivary glands of mosquitoes infected with *PbMLFK*-KO or *Pb*A-WT parasites. We found a 4-fold difference in the number of *PbMLFK*-KO sporozoites compared with *Pb*A-WT sporozoites (Fig. 4e). The *PbMLFK*-KO sporozoite morphology and other parameters were similar to those of *Pb*A-WT sporozoites. Because *Pb*MLFK does not express in the ookinete and zygote stages, we did not check for the *PbMLFK*-KO phenotype during these stages. Taken together, these results indicate that *Pb*MLFK plays an important function during oocyst development in the mosquito midgut.

### *PbMLFK*-KO parasites are defective in cell traversal and liver-stage growth

Sporozoite migration through the host cell is essential for infection and activates the apical exocytosis of sporozoites, which in turn helps in the productive invasion of host cells. Our findings suggest that *PbMLFK*-KO parasites were defective in their capacity to traverse host cells. *PbMLFK*-KO parasites were two times less efficient in migration through the host cell than were WT parasites (Fig. 4f). We quantified the *PbMLFK*-KO and *Pb*A-WT parasite loads in the livers of the infected mice by quantitative RT-PCR (qRT-PCR) using cDNA templates made from total RNA. The results revealed that there was a 100-fold difference in the parasite 18 S rRNA copy numbers between *PbMLFK*-KO and WT sporozoite-infected mouse livers (Fig. 4g). These findings were further verified using the blood smear test. The data demonstrated that there was a 2 day delay in the appearance of *PbMLFK*-KO parasites in the blood (pre-patent period) compared with WT parasites (Table 1 and Fig. 4h). Multiple publications related to malaria infection has established that one day delay in pre patent period corresponds to ~10 fold decrease in initial liver stage burden. Collectively, these results suggest that *Pb*MLFK plays a crucial role during the liver stage of *P. berghei* parasites.

EEF images and morphometric analyses of EEFs show that the average size of *PbMLFK*-KO parasistes was 2.16 times smaller than that of WT parasites (Fig. 4i,j and k). Based on diameter, the parasite population was divided into three size groups of <20 μm, 20–30 μm and >30 μm. The smallest size group (<20 μm) encompassed only 17% of the WT parasites, whereas it constituted 81% of the *PbMLFK*-KO parasite population (Table 2). The medium size group of 20–30 μm included 63% of the WT population and only 19% of the *PbMLFK*-KO parasite population. The third and largest size group (>30 μm) accounted for 20% of the WT and none for the *PbMLFK*-KO parasite population (Fig. 4l and m). Furthermore, to check the effect of *PbMLFK* gene deletion on the parasite growth during the blood stage, 100,000 *PbMLFK*-KO or WT blood stage parasites were each intravenously injected into five mice. During the blood stage, the multiplication of parasites was monitored by Geimsa-stained thin blood smears. The results showed that the *PbMLFK*-KO parasites had no defect compared with *Pb*A-WT parasites during the erythrocytic cycle (Table S1).

### *Pb*MLFK effect on host gene expression

**Figure 5:**
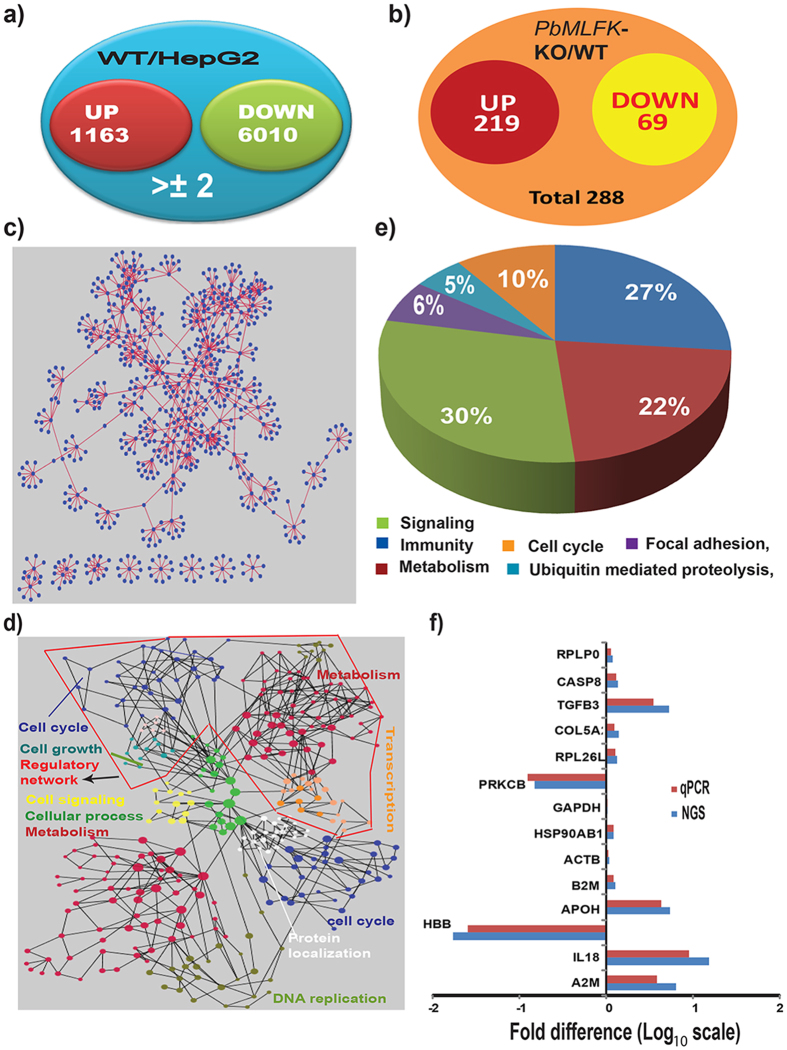
Transcriptome data analysis. (**a**) Venn diagram illustrating the numbers of affected genes in host cells during infection with WT parasites. WT/HepG2: represents the fold change of particular genes in WT infection compared with uninfected HepG2 cells. (**b**) Venn diagram summarizing the significantly affected genes (>2-fold) of the host cell during *PbMLFK*-KO parasite infection compared with WT. *PbMLFK*-KO/WT: represents the fold change of particular genes in *PbMLFK*-KO infection divided by the fold change of that particular gene in WT infection of host cells. (**c**) Network analysis for host genes affected by *PbMLFK*-KO compared with WT parasites, which showed ≥±2-fold change. (**d**) Functional cluster analysis of host genes affected by *PbMLFK*-KO parasites. Nodes represented by the same color are involved in the same biological processes. (**e**) Kyoto Encylopedia of Genes and Genomes (KEGG) pathway analysis of *PbMLFK*-KO-affected host genes compared with WT parasite infection. The host genes showing a fold change of >±2 were considered for pathway analysis. The pie chart shows pathways in which host genes were upregulated during *PbMLFK*-KO parasite infection compared with WT. (**f**) Validation of NGS data by quantitative real-time PCR. ACTB (beta actin) and HSP90AB1 were used as housekeeping genes. Blue and red bars represent the fold change in log scale for host genes analyzed in NGS data and real-time PCR, respectively.

To determine the function of *Pb*MLFK in host, we performed whole transcriptome analysis of host mRNAs collected at 22 hr post sporozoite infection of HepG2 cells. We chose this time point based on a previous microarray study showing the maximum number of gene changes at approximately 24 hr post infection^28^. Using the next generation sequencing (NGS) transcriptome data, we were able to obtain 35,517,455 and 35,678,306 reads for WT and *PbMLFK*-KO infected cells, respectively, which maps to ~16,000 genes of the total 39,904 human genes (GRCH37.P.13). Transcriptome data revealed that approximately 589 genes showed differential expression in *PbMLFK*-KO-infected host cells compared with WT-infected host cells (Table S2). In the case of wild type parasite infection, compared with normal HepG2 cells, the expression of >7,000 host transcripts changed over 2-fold (Fig. 5a). Of the total 589 differentially expressed mRNAs, the levels of 288 mRNA changed more than two-fold, with 219 being upregulated and 69 being downregulated (Fig. 5b). The host cell network of these differentially expressed host genes (>2-fold) is shown in Fig. 5c. Figure 5d shows the cluster analysis of *PbMLFK*-KO-affected host genes. The nodes in one color are involved in same biological processes. Figure 5e illustrates the KEGG pathway analysis of *PbMLFK*-KO-affected host genes compared with WT parasite infection. Pie chart analysis shows that the major pathways affected were associated with signaling, metabolism, immunity, focal adhesion and the cell cycle. NGS transcriptome data were validated by qRT-PCR using independent biological repeats. The list of primers and their sequences, which were used to validate the NGS transcriptome data by qRT-PCR, is shown in Table S3. Figure 5f shows the quantitative PCR (qPCR) results along with the NGS fold change. Quantitative PCR results coincided with NGS data (within a maximum 2-fold deviation). Although the mRNA levels changed, we did not analyze the protein levels and are therefore unable to comment on their levels.

## Discussion

The emergence of drug-resistant *Plasmodium* species warrants an urgent need for novel drug targets to prevent the spread of this parasite. Protein phosphorylation and dephosphorylation regulate various cellular processes, including cell division and signal transduction. In eukaryotes, more than 500 genes encode for protein kinases (PKs) that cover ~2% of all genes. Malaria parasites pass through a series of developmental stages; in different host tissues, this provides distinct surroundings for the developing parasites. Therefore, parasites are likely to have sensing mechanisms to receive the signals generated by altered surroundings in different host tissues. This function might be fulfilled by kinases, which act as a sensor for receiving signals from the surroundings. Some *Plasmodium* PKs have orthologs in mammalian PKs, but the majority of them do not cluster within any known eukaryotic PK families (orphan kinases) or belong to an established PK family and are thus highly divergent (semi-orphan kinases). Divergence between the mammalian and *Plasmodium* kinome^29^ offers an attractive target for novel classes of antimalarial molecules.

In this study, we characterized a protein kinase of *P. berghei* that belongs to the FIKK family of protein kinases. Using bioinformatics tools, we found that the C-terminus of *Pb*MLFK is highly conserved in other *Plasmodium* species. The conserved C-terminus of *Pb*MLFK consists of a kinase domain, an ATP binding site, a substrate-binding site, an active site and an activation loop. *Pb*MLFK is expressed during mosquito and liver stages but not in the blood stage of *P. berghei*. From these studies, we conclude that *Pb*MLFK has a broad expression profile in the *P. berghei* life cycle and may play an important role during these stages. *Pb*MLFK contains two conserved, functional PEXEL motifs in its C-terminus. In the parasitized RBC episomal expression assay^4^,^5^, *Pb*MLFK export was completely abrogated only when both PEXEL motifs were mutated simultaneously, highlighting that both motifs are active. *Pb*MLFK does not contain an obvious signal sequence, and how it is exported across the ER is thus unclear. Bioinformatics analysis showed that *Pb*MLFK contains a transmembrane region towards the end of the protein sequence that could act as a post-translational translocator signal for ER trafficking and export to host cells, as previously reported for PfEMP1^25^. The presence of functional PEXEL motifs indicates that this protein likely plays a role in parasite infection and/or host modulation.

To examine the role of *Pb*MLFK during the life cycle of *P. berghei*, we generated a *PbMLFK*-deficient parasite line. A study by Tewari *et al*.^23^ stated that they were not able to delete this gene; hence, it is likely essential. Tewari *et al*. attempted to disrupt amino acids 947–1123 (of a total 1192 amino acis), which forms the integral part of the kinase domain and is a highly conserved gene region in other species of *Plasmodium*. They designed upstream and downstream targeting sequences from within the gene sequence, which could be refractory to double homologous recombination. This design is likely why Tewari *et al*. was not able to disrupt this gene^23^. We used a different strategy to knock out the gene and successfully deleted it from the parasite genome. We used UTR regions at both ends of the gene for double homologous recombination. Clonal populations were verified for gene deletion and were subsequently used in phenotyping. The parasite loads in the midguts of infected mosquitoes were four-fold less for *PbMLFK*-KO than for the WT parasite. The reason for the reduced parasite load was due to the reduced number of the oocysts. *PbMLFK*-KO parasites also resulted in fewer sporozoites in the salivary glands of infected mosquitoes compared with WT parasites. Previously, it was reported that protein kinase-4 (PK4), a blood stage S/T kinase, regulates the development of the blood stage of *Plasmodium.* PK4 regulates the phosphorylation of elongation factor-2 alpha (EF2α) and inhibits protein synthesis in trophozoites, schizonts and gametocytes^30^. *Pb*MLFK is also a S/T kinase, we expected it to regulate a similar function in liver stage parasites. We analyzed the *PbMLFK-*KO parasite growth in mammalian hosts using a thin blood smear test and found a two-day delay in the pre-patent period in the case of *PbMLFK*-KO sporozoites compared with the WT parasites. In addition, using qPCR, we found a 100-fold reduction in parasite load (18SrRNA copy numbers) in the liver of mice infected with *PbMLFK*-KO parasites compared with those infected with WT parasites. Both results (qPCR and smear test) concur with each other. The above findings indicate that *PbMLFK*-KO parasites were defective in growth during the liver stage and that this protein may thus regulate an important function during this stage. IFA using HepG2 cells infected with *PbMLFK*-KO or WT parasites showed that *PbMLFK*-KO parasites were smaller in size than were WT parasites. The presence of functional PEXEL motifs makes *Pb*MLFK an important FIKK kinase, which might be involved in host modulation and may be necessary for parasite growth and survival.

To better understand the role played by *Pb*MLFK in host cells, we performed mRNA sequencing of host cells infected with *PbMLFK*-KO or *Pb*A-WT parasites. The infected host cell transcriptome data illustrate that *Pb*MLFK significantly affected pathways associated with the immune system, signaling, metabolism, the cell cycle and focal adhesion. The transcript level of IL-18 in host cells was decreased during WT parasite infection but increased during *PbMLFK*-KO parasite infection. IL-18 is a pleiotropic cytokine, which plays a crucial role in the production of IFN-γ from activated macrophages, natural killer (NK) cells and T-cells. It also leads to the secretion of TNF-α and IL-1β from mononuclear monocytes. IL-18 R1 and IL-1R1 were also upregulated over three fold during *PbMLFK*-KO compared to WT parasite infection. IFN-γ is known to inhbit the liverstage parasite growth^31^ thus it makes sense that liver stage parasites try to control IL-18 mediated IFN-γ production by the host. Previous studies have shown that IL-18 plays a protective role against *Plasmodium* infection^32^. The transcript level of IL-11, an anti-inflammatory cytokine of IL-6 family, was increased following WT parasite infection and decreased following *PbMLFK*-KO parasite infection. IL-11 is also involved in the healing of hepatic injury caused by microbial infections^33^,^34^. The wild type parasite on one hand is involved in reducing the proinflammatory (IL-18) and on other hand it also inreases the anti-inflammatory (IL-11) transcripts while *PbMLFK*-KO parasite fails in both the functions. Thus it appears that *PbMLFK* has a role in regulating interleukins that may be deleterious to parasite growth. IL-8, a chemokine, is a chemotactic factor for neutrophils and granulocytes and induces their migration towards the infection site^35^. The transcript level of IL-8 was significantly decreased in *PbMLFK*-KO infection compared with WT parasite infection.

Studies focused on the metabolism of parasitized hosts have also shown that mosquito and blood stage parasites utilize the host amino acids and incorporate them into parasite proteins^36^. Such a requirement has not yet been documented for the liver stage of the malaria parasite. Our data show that the mRNA levels of various proteins involved in amino acid metabolism, namely the amino acids arginine, proline, glycine, serine, threonine, glutathione, phenylalanine and lysine, changed during *PbMLFK*-KO infection. Arginine serves as the source of nitric oxide (NO) in macrophages and endothelial cells^37^,^38^. The metabolism of arginine is highly important from the parasite’s perspective because it serves as the source of N atoms for nitric oxide, which is involved in parasite destruction. Arginine is the most versatile amino acid because it has multiple metabolic fates and can be converted into proline and glutamate.

To summarize, the present study identifies a novel parasite FIKK kinase with functional PEXEL motifs and a broad expression profile throughout the parasite life cycle, from the early oocyst stage in mosquitos to the late liver stage in mammalian hosts, but with a notable absence in the blood stages. *Pb*MLFK plays a crucial role in mosquito and liver stage development of the parasite, its absence modulates several crucial pathways within the infected host cell and offers an ideal target for prophylactic drug development. MLFK is possibly invoved in regulation of Interleukins like IL-18 and IL-11. To our knowledge, this is the first report showing the ability of a kinase to regulate the development of both the stages (mosquito and liver) of malaria parasite.

## Material and Methods

### Ethics statement

All animal work and protocols qualified for an ethical review process and were approved by the NII Institutional Animal Ethics Committee (IAEC). Animal maintenance and experiments were performed in accordance with the Committee for the Purpose of Control and Supervision on Experiments on Animals (CPCSEA) guidelines of the Govt. of India. The IAEC approval number for the project is NII-217/09.

### PCR to check RNA expression in *Plasmodium* life stages

*Pb*A mixed blood stages were obtained from infected mice, Scizonts were purified on a 55% (v/v) Nycodenz^39^, Zygote and Ookinete were obatained as per the protocol described by Rodrigues *et al*.^40^. Oocyst and sporozoite were obtained from parasite infected mosquitoes. EEF were prepared by infecting HepG2 cells with sporozoites and harvesting at indicated time points. RNA was isolated using the Trizol^TM^ reagent (Invitrogen, USA) as per the manufacturers instructions. RNA was converted into cDNA using Accuscript cDNA synthesis kit (Agilent, USA, Cat No. #200820). The 50 μl PCR mixture contained 4 μl cDNA, 50 picomoles each forward [ATGCTAATGATATCTAAACAAAAATAAAAAAAATGT] and reverse primer [TGTGATAAACCTGCATCATG], 1 × PCR buffer containing 2.5 mM MgCl_2_, 0.2 mM dNTPs and 1.5 U Taq polymerase. Cycling conditions were 95 °C for 3 min × 1 cycle, 5 cycle of 95 °C for 15 sec-45 °C for 20 sec-68 °C for 2 min and 25 cycle of 95 °C for 15 sec-55 °C for 25 sec-68 °C for 2 min. The expected PCR product size is 932 bp.

### Immunofluorescence assay (IFA) of the oocyst stage

Female *Anopheles* mosquitoes were allowed to feed on *Pb*A infected mice, and the midguts of the infected mosquitoes were then excised at 4, 7, 10 and 12 days post blood meal. The midguts were fixed in 4% paraformaldehyde for 10 minutes at room temperature. The midguts were then permeabilized with 0.2% saponin for 20 minutes and blocked with 3% BSA for 1 hr at room temperature. IFA was performed using an anti-*Pb*MLFK peptide mouse polyclonal antibody at a 1:500 dilution for 90 minutes at room temperature. Goat anti-mouse Alexa 488-conjugated secondary antibody (dilution 1:1000) was used to track *Pb*MLFK in the oocyst stage of *P. berghei*. IFA of salivary gland sporozoites, liver stages and blood stages were done in similar manner and the details are provided in supplemental methods.

### Episomal transfection for PEXEL motif functional assays

*Pb*A schizonts were purified from parasite-infected blood using the Nycodenz method as per the protocol described earlier^39^. The vector used for transfection is a modified vector which we termed as pEpi (details in supplementary methods). The *PbMLFK* C-terminal sequence containing 1491 nucleotides (including the PEXEL coding sequence) was cloned in pEpi vector between *Kpn*I and *Xho*I sites in frame with GFP (at the C-terminus). The primers used for PCR amplification of the *PbMLFK* C-terminus were IDL1-FP and IDL2-RP. This construct was named pEpi-PbMLFK. Electroporations were performed as described by Singh *et al*.^6^. Blood was taken from the tail of injected mice, and transfected parasites were examined for *Pb*MLFK-GFP fusion protein export using a Zeiss fluorescence microscope (Axio-Imager M2).

Next, we mutated the two putative PEXEL motifs to identify the functional motif present in *Pb*MLFK. The first and third amino acid residues in the *Pb*MLFK PEXEL motif were replaced by alanine (A). The first PEXEL motif in *Pb*MLFK is KLLHS (P1), which was mutated to ALAHS. The specific primers used to mutate the *Pb*MLFK P1-motif were IDL3-FP and IDL4-RP. These primers were designed in such a way that the mutation would generate a *Hae*-III restriction site that was not present in the WT *PbMLFK* gene and was used for the screening of mutants. The second PEXEL motif in *Pb*MLFK is KILYE (P2), which was mutated to AIAYE. The primers used for mutagenesis of the P2-motif were IDL5-FP and IDL6-RP. Mutation of both PEXEL motifs was confirmed by nucleotide sequencing. All of the primers used are described in Table S3.

### Parasite transfection for generating *PbMLFK*-deficient parasites (*PbMLFK*-KO)

To generate the *PbMLFK* knockout we prepared a targeting construct in the knockout vector [pBC-GFP-hDHFR-Asc] by cloning the two PCR products 5′ UTR (890 bp) and 3′UTR (781 bp) of the *PbMLFK*. The targeting construct contained the *hDHFR* gene for drug selection and GFP for microscopic analysis of transfected parasites. *PbMLFK*-3′UTR was amplified by PCR using the following primers: IDL7-FP with an *Xho*I restriction site and IDL8-RP with a *Cla*I restriction site. *PbMLFK* 5′UTR was similarly amplified using primers IDL9-FP, containing a *Not*I restriction site, and IDL10-RP, containing a *Sac*II restriction site. The targeting construct was prepared using an Endo-free plasmid midi prep kit (Qiagen). The targeting construct was linearized with *Sca*I restriction enzyme and electroporated using an amaxa nucleofector device (Nucleofector-II) into the WT-*Pb*A schizonts. Electroporation, selection and dilution cloning were performed as per the method described in Singh *et al*.^6^. The primers used are described in Table S3.

### Southern hybridization to confirm *PbMLFK* gene deletion in the *Pb*A parasite

Southern blot analysis was performed using a digoxigenin (DIG) high prime DNA labeling and detection kit (Roche, Mannheim, Germany). Briefly, genomic DNA of transformed parasites and WT-*Pb*A was digested with *Acl*I and *Hind*III. Digested DNA was separated on a 1% agarose gel at 40 V for 6 hr and then transferred overnight by a capillary method to a positively charged Nylon membrane (Roche, Germany). Transferred DNA was UV cross-linked to the Nylon membrane at a setting of 1200 mJ/cm^2^. *PbMLFK*-3′UTR PCR product (781 bp) was used as a probe. The probe was prepared according to a protocol given in the Roche DIG high prime DNA labeling and detection kit.

### Phenotypic characterization of *PbMLFK*-KO in mosquito and blood stages of the malaria parasite

*Anopheles stephensi* mosquitoes (3–5 days old, female) were infected with *PbMLFK*-KO or WT-*Pb*A parasites via blood feeding for 10 minutes on parasite-infected mice having a ~0.5% gametocyte count. The infected mosquitoes were maintained at 22 °C and 80% humidity under a cyclic condition of 12 hr light/12 hr dark. Infected mosquitoes were analyzed on day 14 for oocyst counts and on day 18 for numbers of salivary gland sporozoites.

### Genomic DNA isolation from infected midgut

Oocyst positive midguts were used for genomic DNA isolation using a method described previously^41^. Details can be found in supplemental methods.

### *In vivo* infectivity assay

Five C57BL/6 mice were intravenously injected with 6000 sporozoites from WT-*Pb*A or *PbMLFK*-KO parasites. Parasite 18 S rRNA load in the liver was measured as described in Singh *et al*.^6^. In another set of experiments, 5 BALB/c mice from both groups were intravenously (i.v.) injected with 6000 sporozoites of WT-*Pb*A or *PbMLFK*-KO parasite. We later followed the emergence of parasite in Geimsa-stained blood smears. The time lag between sporozoite inoculation and the first detection of parasite in the blood is termed the pre-patent period. One day delay in pre-patent period reflects 10 fold reduction in initial liver stage burden. Similarly a 2-day delay corresponds to 100 fold reduction in initial liver stage burden.

### *In vitro* cell traversal assay

Sporozoite migration through host cells was quantified by counting the wounded host cells caused by sporozoite traversal in the presence of cell-impermeable FITC-dextran as per the protocol described by Mota, M. M. *et al*.^42^.

### *In vitro PbMLFK*-KO parasite size measurements

We initiated infection of 70–80% confluent HepG2 cells in 24-well tissue culture plates with 2.5 × 10^4^ sporozoites. IFA was performed in a manner similar to that of Singh *et al*.^6^. Images of parasites were taken using a 63× oil objective in an Axio-imager M2 (Zeiss) and processed for area and diameter measurements using Axiovision automeasure software (Zeiss).

### Kinase activity assay

Kinase activity was assayed in IP (immunoprecipitaion) elutes (obtained from 150 infected mosquito midguts) and the C-terminal recombinant *Pb*MLFK protein (GST column purified, 500 ng/assay), using a phospho-serine/threonine ELISA-based assay kit (Upstate cell signaling solutions). Kinase assays were performed according to the manufacturer’s instructions. Details of protein preparation are given in supplementary methods.

### Cell infection, cell sorting, RNA isolation, deep cDNA sequencing and data analysis

WT-GFP or *PbMLFK*-KO sporozoites (2 × 10^6^ isolated from the salivary gland of infected mosquitoes) were added to the HepG2 cells (1 × 10^6^ cells/well in 6-well plates) to initiate infection. The remainder of the method was performed as described by Jaijyan *et al*.^43^.

### Validation of target genes in NGS by real-time PCR

To validate the next generation sequencing data, we performed real-time PCR for the selected genes using SYBR Green PCR Master Mix (Life Technologies, India) on a 2 × 2 realplex detection system (Eppendrof, Germany). The primers for the selected genes (Table S3) used in real-time PCR were designed using primer BLAST software (NCBI). Primers detailed in Table S3 were obtained from Sigma (India). The HSP90AB and ACTB primers were used as housekeeping controls for all samples. The real-time PCR reaction conditions were as follows: one cycle at 95 °C for 2 minutes and 40 cycles at 95 °C for 30 seconds, 55 °C for 20 seconds and 68 °C for 30 seconds, followed by a melting curve. For each sample, the real-time PCR reaction was performed in triplicate 20 μl reaction volumes. The relative expression or fold change for each selected gene between WT-*Pb*A and *PbMLFK-*KO was determined using the 2^**−**ΔΔCt^ method.

### Host cell network analysis

was performed as described by Jaijyan *et al*.^43^. Details are provided in supplemantal methods^44^,^45^,^46^.

## Additional Information

**How to cite this article**: Jaijyan, D. K. *et al*. A novel FIKK kinase regulates the development of mosquito and liver stages of the malaria. *Sci. Rep.* **6**, 39285; doi: 10.1038/srep39285 (2016).

**Publisher's note:** Springer Nature remains neutral with regard to jurisdictional claims in published maps and institutional affiliations.

## Supplementary Material

Supplementary Information

## Figures and Tables

**Table 1:** The delay in pre-patent period in C57BL/6 mice infected with *PbMLFK*-KO compared with WT sporozoites.

Genotype	Dose	No. of mice injected	% of patent mice	Pre patent day	Delay in patency (no. of days)
*PbA*-WT	6000	5	100	3	0
*PbMLFK-*KO	6000	5	100	5	2

Dose represents the number of sporozoites injected intravenously (iv.) per mouse.

**Table 2:** *PbMLFK*-KO parasites are defective in growth during liver stage, as compared to WT parasites.

↓ Host	Parasite→	Size group	*PbA* Wild type		*PbMLFK* knockout	
			Diameter (SD)	Area (SD)	Diameter (SD)	Area (SD)
		<20	18.64 (1.2)	274.17 (37.68)	13.96 (2.79)	158.17 (63.38)
Mouse C57BL/6		20–30	24.53 (3.1)	480.45 (125.88)	24.02 (2.8)	458.72 (104.63)
		>30	35.06 (3.05)	972.00 (171.46)	0.0	0.0

The average values of area and diameter of *PbMLFK*-KO and WT parasites, which was measured in the liver of C57BL/6 mice 48 hr after infection with the respective parasites. Based on size, the *PbMLFK*-KO and WT parasite populations were divided into three size groups.

Unit of diameter and SD in brackets are in micrometer (μm) and unit of area and SD in brackets are in square of micrometer (μm)^2^.
