# Supplementary material for: A novel FIKK kinase regulates the development of mosquito and liver stages of the malaria
Source: Sci Rep. 2016 Dec 20;6:39285. doi: 10.1038/srep39285 (PMC5172157; doi:10.1038/srep39285)

**A novel FIKK kinase regulates the development of mosquito and liver stages of the malaria.**

Dabbu Kumar Jaijyan1, Praveen Kumar Verma2, Agam Prasad Singh1*
1 Infectious Diseases Laboratory, National Institute of Immunology, Aruna Asaf Ali Marg, New Delhi-110067, India
2 Plant Immunity Laboratory, National Institute of Plant Genome Research, Aruna Asaf Ali Marg, New Delhi-110067, India
* Corresponding author: Agam P. Singh,

## Supplementary Methods, Tables and Figures:

## 1. Supplementary methods:

**Animals and Parasites:** The animals used in this study were six- to eight-week-old male and female CD1, BALB/c or C57BL/6 mice or 60-120 g SD1 rats and were acquired from the institution's animal facility. Mice were used for transferring the parasite life cycle between mosquitoes and rodents. Infected mosquitoes were allowed to feed on sugar pads and were maintained at 25°C and 70% relative humidity until the sporozoite dissection. Sporozoites were isolated from the salivary glands of infected mosquitoes after 18 days of feeding on blood meal. Infected mosquitoes were first rinsed with 50% ethanol and then washed with RPMI media. Salivary glands of infected mosquitoes were dissected in RPMI media with 10% FBS, and sporozoites were obtained by gentle grinding of dissected salivary glands followed by centrifugation in a microfuge at 800 rpm for 3 minutes. Sporozoites numbers per µl of sample were determined by counting in a hemocytometer. To know per mosquito sporozoite yield, total number of sporozoites obtained was divided by number of mosquitoes dissected.

**IFA of salivary gland sporozoites**: WT sporozoites were isolated from the salivary glands of infected mosquitoes and coated overnight at 4°C on twelve chambered Teflon-coated slides (TEKDON Inc., Florida, USA). Sporozoites were fixed, permeabilized, blocked and treated with primary antibody in the same way as for oocysts. Goat anti-mouse Alexa 488-

conjugated secondary antibody (dilution 1:2500) was used to track *Pb*MLFK in *P. berghei* sporozoites. Nuclei were stained with DAPI (4, 6-diamidino-2-phenylindole).

**IFA of the liver stage of *P. berghei*:** HepG2 cells were grown on circular 15 mm cover slips in a 24-well plate containing DMEM with 10% FBS and were maintained at 37°C and 5% $CO_2$. Subconfluent HepG2 cells in each well were infected with 10,000 *Pb*A sporozoites isolated from the salivary glands of infected mosquitoes in RPMI with 20% FBS. Infected HepG2 cells were harvested at 6, 12, 24, 36 and 48 hpi, fixed with 4% para-formaldehyde (EMS, Hatfield, PA, USA) for 10 minutes and permeabilized with 0.2% saponin in PBS for 15 minutes. Cells were blocked with 3% BSA in PBS (Sigma, India) for 1 hr at room temperature. *Pb*A-infected cells were incubated with mouse anti-*Pb*MLFK peptide antibody (1:1000) for 2 hrs at room temperature. Cells were washed three times with PBS and incubated with goat anti-mouse IgG Alexa 488 secondary antibody (1:1000) for 1 hr at room temperature, followed by three washes with PBS. Stained cells were mounted on glass slides along with anti-fade reagent and imaged with the help of a fluorescence microscope (Zeiss, Axio imager M2).

**IFA of the blood stage:** On a glass slide, thin smears of blood with 3-4% parasitemia were prepared, fixed with methanol and permeabilized with 0.2% saponin for 10 minutes at room temperature. The remainder of the procedure was performed as above in the case of sporozoite IFA.

**Construction of pEpi Vector:** A cassette which was amplified from the vector pL0016 using specific primers [EPI-1, 5’-GGCGGCCGCCAATTTCGATATCGAA TTCC-3’ and EPI-2, 5’-GGGCGCCTGGGCTTTAACTTCC-3’] having *Not*I and *Sac*II site, was introduced by TA cloning method in pCR2.1-TOPO vector. The cassette contained the EF1α promoter➔ *GFP* sequence➔ *PbDHFR* 3’-UTR. In the cloned vector, GFP ATG

codon was replaced with KpnI-XhoI restriction site by mutagenesis using primers EPI-3, 5'CATAGGGGGATCCGGTACCCTCGAGAGTAAAGGAGAAGAAC-3' and EPI-4, which was reverse complement of EPI-3. This final vector we termed as pEpi.

**Genomic DNA isolation from infected midgut:** Mosquito midguts (WT-*Pb*A and *PbMLFK*-KO) were dissected in RPMI media on day-10 post blood meal infection and washed with 1X PBS. Only oocyst positive midguts were used for genomic DNA isolation using a method described previously (1). The purity of genomic DNA was determined by measuring ratio of the OD 260/280. Genomic DNA (500 pg) was used as a template in real-time PCR using parasite 18S rRNA gene primers to check for parasite load at the oocyst stage in mosquitoes. The exact numbers of oocysts for WT-*Pb*A and *PbMLFK*-KO parasites in mosquito midguts were determined by IFA using monoclonal *Pb*-CSP-antibody (3D11). For this procedure, 30 midguts were excised from infected mosquitoes and processed for IFA using monoclonal 3D11 [anti-CSP antibody], followed by counting using a fluorescence microscope (Axio-Imager M2, Zeiss).

**Details of protein used in Kinase assay:** An IP lysate containing full-length *Pb*MLFK was obtained by incubating anti-*Pb*MLFK peptide antibody (peptide sequence 'KCSDPLQED' conjugated to KLH) with WT-*Pb*A parasite-infected mosquito midgut lysate. The precipitated *Pb*MLFK protein was purified using protein-A agarose beads and used in kinase activity assays. *Pb*MLFK C-terminal recombinant GST-tagged protein having a kinase domain was purified on pre-packed columns containing GST binding beads (GE Health Sciences). For expression, the codon optimized (for *E. coli*) C-terminus of PbMLFK was cloned into pGEX6P1. Protein was expressed in *E. coli* BL21 cells (RIL codon plus) after induction with 0.5 mM IPTG at 30°C for 12 hr, and induction occurred when $A_{600}$ reached a value of 0.5. Protein was purified following standard protocol recommended by

GST beads supplier (GE Health sciences). The eluted protein was ~90% pure. Protein was stored at -80°C in 10% glycerol until used.

**Host cell network analysis:** The differentially expressed host genes showing more than a ± 2-fold difference and with a p value <0.05 were selected for the pathway analysis using GeneCodis3 software (2). The gene ontology (GO) analysis of selected genes in each pathway was completed with the help of the Bingo 2.3 plugin in CytosCape 2.8.3 (3). The pathway-specific host genes were used to build a host cell network modulated by the *PbMLFK*-KO parasite. For this procedure, each gene in a specific pathway was searched in the STRING database (http://string.embl.de) for its direct interacting partners. STRING is a database of both known and predicted protein-protein interactions. All of the interactions/associations showing a combined score of >0.7 were selected for each node and used to build the network. Scoring in the STRING database represents the confidence limit for each described interaction or association. A combined score of 0.7 represented a high stringency criterion. The associations for all of the *Pb*MLFK-affected host genes in one pathway were merged together and imported into Cytoscape 2.8.3 as a two-column network (3). The network analysis was performed with the help of the Cytoscape plugin Network Analyzer 2.7 (4).

**References:**

1. Weiss, M.M. & Vanderberg, J.P. (1976) Studies on Plasmodium ookinetes. 1. Isolation and concentration from mosquito midguts. J Protozool **23**, 547-51

2. Tabas-Madrid, D. et al. (2012) GeneCodis3: a non-redundant and modular enrichment analysis tool for functional genomics. Nucleic Acids Res **40,** 478-483

3. Cline, M.S. et al. (2007) Integration of biological networks and gene expression data using Cytoscape. Nat Protoc **2,** 2366- 2382

4. Assenov, Y. et.al. (2008) Computing topological parameters of biological networks. Bioinformatics **24**, 282-284

## 2. Supplementary Tables:

**Table S1.** ***PbMLFK*-KO parasite is normal during the blood stage growth**

| Genotype | Dose | No. of mice injected | % of patent mice | Prepatent day | Delay in prepatent day |
|---|---|---|---|---|---|
| *Pb*A-WT | 100000 | 5 | 100 | 1 | 0 |
| *PbMLFK*-KO | 100000 | 5 | 100 | 1 | 0 |

Dose represents the number of infetced RBC injected intravenously (i.v.) per mouse

**Table S1:** No delay in the pre-patent period in BALB/c mice when infected with *PbMLFK*-KO blood stage parasites compared with WT parasites. Doses represent the number of infected RBC injected intravenously (i.v.) per mouse.

## Table S2

**List of host transcript levels that changed due to PbMLFK-KO infection compared to Wild Type parasite infection**

| | PbMLFK-KO/ WT | | Wild Type PbA infected HepG2 | | | | PbMLFK-KO [Pb122500-ko] infected HepG2 | | | |
|---|---|---|---|---|---|---|---|---|---|---|
| Gene Name | Fold Change | P-value | Total gene reads | RPKM | Chromosome | Chromosome region start | Total gene reads | RPKM | Chromosome | Chromosome region start |
| IL18 | **15.43** | **0.04** | 18.00 | 0.40 | NC_000011 | 112013974 | 660 | 6.2 | NC_000011 | 112013974 |
| FAM72D | **12.65** | **0.03** | 32 | 0.55 | NC_000001 | 143896452 | 624 | 7 | NC_000001 | 143896452 |
| NPIPB3 | **7.41** | **0.00** | 156 | 2.06 | NC_000016 | 21413455 | 2265 | 15.3 | NC_000016 | 21413455 |
| ALB | **7.22** | **0.00** | 123 | 2.56E+00 | NC_000004 | 74269972 | 1406 | 18.49 | NC_000004 | 74269972 |
| EME1 | **6.78** | **0.00** | 191 | 3.11E+00 | NC_000017 | 48450581 | 2237 | 21.05 | NC_000017 | 48450581 |
| RELN | **6.77** | **0.01** | 866 | 1.69E+00 | NC_000007 | 103112231 | 5682 | 11.46 | NC_000007 | 103112231 |
| LOC728728 | **6.69** | **0.03** | 33 | 1.38 | NC_000001 | 228154712 | 421 | 9.22 | NC_000001 | 228154712 |
| A2M | **6.51** | **0.03** | 140 | 1.5 | NC_000012 | 9220304 | 1547 | 9.74 | NC_000012 | 9220304 |
| AFP | **6.22** | **0.00** | 4929 | 137.5 | NC_000004 | 74301933 | 59413 | 855.84 | NC_000004 | 74301933 |
| DKK1 | **5.80** | **0.00** | 333 | 9.95 | NC_000010 | 54074041 | 3568 | 57.72 | NC_000010 | 54074041 |
| ERCC5 | **5.70** | **0.05** | 126 | 1.45 | NC_000013 | 103498191 | 1418 | 8.28 | NC_000013 | 103498191 |
| VTN | **5.63** | **0.01** | 78 | 2.47 | NC_000017 | 26694298 | 790 | 13.9 | NC_000017 | 26694298 |
| APOH | **5.56** | **0.00** | 100 | 3.74 | NC_000017 | 64208147 | 910 | 20.8 | NC_000017 | 64208147 |
| SEC14L6 | **5.53** | **0.05** | 210 | 1.48E+00 | NC_000022 | 30920629 | 1412 | 8.18 | NC_000022 | 30920629 |
| TF | **5.46** | **0.00** | 4689 | 9.03E+01 | NC_000003 | 133464977 | 48093 | 492.74 | NC_000003 | 133464977 |
| B3GNT1 | **5.31** | **0.04** | 61 | 1.7 | NC_000011 | 66112843 | 620 | 9.01 | NC_000011 | 66112843 |
| NREP | **5.29** | **0.03** | 206 | 2.03 | NC_000005 | 111065000 | 1802 | 10.73 | NC_000005 | 111065000 |
| FGL1 | **5.22** | **0.00** | 1798 | 7.57E+00 | NC_000008 | 17721894 | 16596 | 39.49 | NC_000008 | 17721894 |
| C2orf72 | **5.16** | **0.01** | 201 | 2.96 | NC_000002 | 231902281 | 2101 | 15.25 | NC_000002 | 231902281 |
| TMEM194A | **5.05** | **0.01** | 327 | 3.14 | NC_000012 | 57449426 | 3304 | 15.83 | NC_000012 | 57449426 |
| FANCD2 | **5.02** | **0.04** | 307 | 1.91 | NC_000003 | 10068113 | 2442 | 9.57 | NC_000003 | 10068113 |
| NHLRC3 | **4.96** | **0.01** | 342 | 3.72 | NC_000013 | 39612448 | 3086 | 18.48 | NC_000013 | 39612448 |
| CENPF | **4.87** | **0.02** | 549 | 2.55 | NC_000001 | 214776522 | 4706 | 12.43 | NC_000001 | 214776522 |
| NPNT | **4.82** | **0.00** | 587 | 4.05 | NC_000004 | 106816588 | 3735 | 19.51 | NC_000004 | 106816588 |
| ATP6AP1L | **4.78** | **0.04** | 90 | 2.2 | NC_000005 | 81601166 | 807 | 10.53 | NC_000005 | 81601166 |
| GEN1 | **4.71** | **0.01** | 539 | 3.31 | NC_000002 | 17935152 | 4649 | 15.61 | NC_000002 | 17935152 |
| TOP2A | **4.68** | **0.00** | 657 | 5.85 | NC_000017 | 38544773 | 5675 | 27.35 | NC_000017 | 38544773 |
| LOX | **4.55** | **0.00** | 494 | 4.44 | NC_000005 | 121398890 | 4298 | 20.21 | NC_000005 | 121398890 |
| TNFRSF19 | **4.48** | **0.00** | 1515 | 11.06 | NC_000013 | 24144509 | 11194 | 49.58 | NC_000013 | 24144509 |
| SLC13A5 | **4.40** | **0.01** | 307 | 4.45 | NC_000017 | 6588038 | 3342 | 19.56 | NC_000017 | 6588038 |
| CENPK | **4.35** | **0.03** | 164 | 2.83 | NC_000005 | 64813593 | 1522 | 12.3 | NC_000005 | 64813593 |
| GPR126 | **4.34** | **0.01** | 686 | 4.1 | NC_000006 | 142623056 | 4771 | 17.79 | NC_000006 | 142623056 |
| GINS1 | **4.34** | **0.01** | 324 | 3.87 | NC_000020 | 25388323 | 2337 | 16.8 | NC_000020 | 25388323 |
| FAM111A | **4.33** | **0.04** | 250 | 2.57 | NC_000011 | 58910219 | 2241 | 11.15 | NC_000011 | 58910219 |
| TSSK3 | **4.25** | **0.05** | 145 | 2.5 | NC_000001 | 32818365 | 1185 | 10.61 | NC_000001 | 32818365 |
| LGR5 | **4.13** | **0.01** | 563 | 4.97E+00 | NC_000012 | 71832931 | 4167 | 20.53 | NC_000012 | 71832931 |
| NRP1 | **4.11** | **0.04** | 796 | 2.76E+00 | NC_000010 | 33466419 | 3352 | 11.36 | NC_000010 | 33466419 |
| SERPINA7 | **4.04** | **0.00** | 245 | 7.44 | NC_000023 | 105277190 | 1765 | 30.03 | NC_000023 | 105277190 |
| LDLRAD1 | **4.04** | **0.01** | 220 | 4.65 | NC_000001 | 54472971 | 2221 | 18.81 | NC_000001 | 54472971 |
| MTR | **3.99** | **0.02** | 1019 | 3.97 | NC_000001 | 236958581 | 6405 | 15.85 | NC_000001 | 236958581 |
| SFXN3 | **3.96** | **0.00** | 1025 | 15.80 | NC_000010 | 102790996 | 8928 | 62.57 | NC_000010 | 102790996 |
| ZNF700 | **3.96** | **0.03** | 214 | 3.53E+00 | NC_000019 | 12035883 | 1737 | 14 | NC_000019 | 12035883 |
| MBNL3 | **3.94** | **0.00** | 2906 | 7.18 | NC_000023 | 131503342 | 13686 | 28.33 | NC_000023 | 131503342 |
| SCML1 | **3.94** | **0.01** | 338 | 5.10E+00 | NC_000023 | 17755591 | 2672 | 20.12 | NC_000023 | 17755591 |
| CDK2 | **3.89** | **0.03** | 164 | 3.46E+00 | NC_000012 | 56360556 | 1227 | 13.45 | NC_000012 | 56360556 |
| C1RL | **3.77** | **0.04** | 296 | 3.33 | NC_000012 | 7246000 | 2316 | 12.57 | NC_000012 | 7246000 |
| AHSG | **3.73** | **0.00** | 2183 | 78.03 | NC_000003 | 186330850 | 17027 | 291.12 | NC_000003 | 186330850 |
| SERPINA3 | **3.72** | **0.00** | 4971 | 152.69 | NC_000014 | 95078639 | 35491 | 568.34 | NC_000014 | 95078639 |
| MRPS25 | **3.72** | **0.05** | 642 | 3.19 | NC_000003 | 15083758 | 4427 | 11.89 | NC_000003 | 15083758 |
| CCNB2 | **3.71** | **0.01** | 269 | 5.87 | NC_000015 | 59397284 | 1372 | 21.77 | NC_000015 | 59397284 |
| EZH1 | **3.71** | **0.03** | 446 | 3.72 | NC_000017 | 40852293 | 2452 | 13.83 | NC_000017 | 40852293 |
| UNC5CL | **3.69** | **0.02** | 278 | 4.36 | NC_000006 | 40994640 | 2248 | 16.11 | NC_000006 | 40994640 |
| PROS1 | **3.68** | **0.02** | 551 | 4.84 | NC_000003 | 93591881 | 2613 | 17.81 | NC_000003 | 93591881 |
| FAM83D | **3.63** | **0.02** | 279 | 4.46 | NC_000020 | 37554955 | 1695 | 16.19 | NC_000020 | 37554955 |
| APOA5 | **3.59** | **0.05** | 117 | 3.37 | NC_000011 | 116660086 | 798 | 12.1 | NC_000011 | 116660086 |
| BUB1 | **3.58** | **0.03** | 354 | 4.04 | NC_000002 | 111395275 | 2225 | 14.47 | NC_000002 | 111395275 |
| NKD1 | **3.57** | **0.00** | 4729 | 11.09 | NC_000016 | 50582241 | 15612 | 39.64 | NC_000016 | 50582241 |
| SPP1 | **3.52** | **0.00** | 2753 | 83.98 | NC_000004 | 88896802 | 18041 | 295.97 | NC_000004 | 88896802 |
| PLK1 | **3.52** | **0.04** | 179 | 4.03 | NC_000016 | 23690201 | 1215 | 14.21 | NC_000016 | 23690201 |
| MGME1 | **3.51** | **0.00** | 521 | 7.5 | NC_000020 | 17949534 | 4375 | 26.35 | NC_000020 | 17949534 |
| APOL1 | **3.51** | **0.03** | 268 | 4.54 | NC_000022 | 36649117 | 1899 | 15.92 | NC_000022 | 36649117 |
| CPVL | **3.50** | **0.00** | 486 | 7.89 | NC_000007 | 29035247 | 2563 | 27.66 | NC_000007 | 29035247 |

| | | | | | | | | | | |
|---|---|---|---|---|---|---|---|---|---|---|
| KNTC1 | **3.50** | **0.05** | 547 | 3.62E+00 | NC_000012 | 123011796 | 3632 | 12.69 | NC_000012 | 123011796 |
| AURKA | **3.49** | **0.00** | 379 | 7.57 | NC_000020 | 54944445 | 2880 | 26.42 | NC_000020 | 54944445 |
| NUSAP1 | **3.47** | **0.01** | 366 | 6.71E+00 | NC_000015 | 41624892 | 2283 | 23.29 | NC_000015 | 41624892 |
| ADD3 | **3.45** | **0.01** | 960 | 7.52 | NC_000010 | 111765627 | 5401 | 25.92 | NC_000010 | 111765627 |
| FGA | **3.42** | **0.01** | 507 | 7.42 | NC_000004 | 155504280 | 3317 | 25.37 | NC_000004 | 155504280 |
| ATAD2 | **3.42** | **0.02** | 852 | 5.87 | NC_000008 | 124332090 | 4448 | 20.07 | NC_000008 | 124332090 |
| MCM8 | **3.42** | **0.04** | 370 | 4.26 | NC_000020 | 5931298 | 2348 | 14.57 | NC_000020 | 5931298 |
| LIPA | **3.41** | **0.04** | 241 | 4.44 | NC_000010 | 90973326 | 1435 | 15.14 | NC_000010 | 90973326 |
| METTL3 | **3.38** | **0.01** | 376 | 6.54 | NC_000014 | 21966282 | 2089 | 22.1 | NC_000014 | 21966282 |
| BUB1B | **3.36** | **0.01** | 589 | 6.91 | NC_000015 | 40453210 | 3418 | 23.24 | NC_000015 | 40453210 |
| PSRC1 | **3.33** | **0.02** | 217 | 5.62 | NC_000001 | 109822176 | 1457 | 18.73 | NC_000001 | 109822176 |
| FAM178A | **3.31** | **0.01** | 1226 | 7.96 | NC_000010 | 102672326 | 8099 | 26.34 | NC_000010 | 102672326 |
| DSN1 | **3.30** | **0.00** | 492 | 8.56 | NC_000020 | 35380194 | 3555 | 28.28 | NC_000020 | 35380194 |
| APP | **3.30** | **0.01** | 906 | 7.66 | NC_000021 | 27252861 | 3662 | 25.27 | NC_000021 | 27252861 |
| SOS1 | **3.30** | **0.02** | 6512 | 6.15 | NC_000002 | 39208690 | 9960 | 20.29 | NC_000002 | 39208690 |
| ZNF334 | **3.30** | **0.04** | 429 | 4.64 | NC_000020 | 45128269 | 2968 | 15.28 | NC_000020 | 45128269 |
| DLX1 | **3.25** | **0.05** | 192 | 4.32 | NC_000002 | 172950208 | 1262 | 14.05 | NC_000002 | 172950208 |
| SCRN1 | **3.23** | **0.04** | 721 | 4.95 | NC_000007 | 29959719 | 3268 | 16.01 | NC_000007 | 29959719 |
| GATAD1 | **3.20** | **0.01** | 676 | 7.05 | NC_000007 | 92076762 | 4514 | 22.52 | NC_000007 | 92076762 |
| ITM2B | **3.19** | **0.00** | 522 | 14 | NC_000013 | 48807274 | 3051 | 44.63 | NC_000013 | 48807274 |
| LYZ | **3.18** | **0.04** | 133 | 5.05 | NC_000012 | 69742134 | 837 | 16.08 | NC_000012 | 69742134 |
| FN1 | **3.17** | **0.00** | 22711 | 123.58 | NC_000002 | 216225177 | 127437 | 391.89 | NC_000002 | 216225177 |
| SUGP2 | **3.17** | **0.04** | 726 | 5.09 | NC_000019 | 19101697 | 3899 | 16.1 | NC_000019 | 19101697 |
| PIGZ | **3.15** | **0.00** | 889 | 15.87 | NC_000003 | 196673214 | 6569 | 50.02 | NC_000003 | 196673214 |
| SYTL2 | **3.15** | **0.00** | 3228 | 12.97 | NC_000011 | 85405264 | 19309 | 40.92 | NC_000011 | 85405264 |
| GPC3 | **3.14** | **0.01** | 6049 | 9.09 | NC_000023 | 132669773 | 12026 | 28.55 | NC_000023 | 132669773 |
| UACA | **3.13** | **0.00** | 3039 | 16.57 | NC_000015 | 70946893 | 16984 | 51.91 | NC_000015 | 70946893 |
| NUF2 | **3.13** | **0.01** | 338 | 7.32 | NC_000001 | 163291723 | 2410 | 22.93 | NC_000001 | 163291723 |
| FBLN1 | **3.13** | **0.04** | 685 | 5.44 | NC_000022 | 45898719 | 3362 | 17.01 | NC_000022 | 45898719 |
| TPX2 | **3.12** | **0.01** | 794 | 9.14 | NC_000020 | 30326904 | 4270 | 28.49 | NC_000020 | 30326904 |
| PEG3 | **3.11** | **0.00** | 6087 | 27.95 | NC_000019 | 57285730 | 33596 | 86.83 | NC_000019 | 57285730 |
| SLC47A1 | **3.11** | **0.01** | 645 | 8.73 | NC_000017 | 19437167 | 4114 | 27.16 | NC_000017 | 19437167 |
| LBR | **3.09** | **0.04** | 452 | 5.32 | NC_000001 | 225589204 | 2574 | 16.45 | NC_000001 | 225589204 |
| CLSTN3 | **3.07** | **0.05** | 535 | 4.78 | NC_000012 | 7281681 | 2764 | 14.67 | NC_000012 | 7281681 |
| PRCP | **3.06** | **0.05** | 293 | 4.92 | NC_000011 | 82535409 | 1279 | 15.06 | NC_000011 | 82535409 |
| PTK7 | **3.04** | **0.02** | 859 | 7.45 | NC_000006 | 43044006 | 4022 | 22.62 | NC_000006 | 43044006 |
| LGALS3BP | **3.03** | **0.00** | 1973 | 48.78 | NC_000017 | 76967335 | 11303 | 147.7 | NC_000017 | 76967335 |
| HMGA2 | **3.02** | **0.05** | 626 | 4.98 | NC_000012 | 66218240 | 3531 | 15.05 | NC_000012 | 66218240 |
| SMC4 | **3.01** | **0.04** | 714 | 5.62 | NC_000003 | 160117078 | 3760 | 16.91 | NC_000003 | 160117078 |
| FGB | **2.99** | **0.00** | 745 | 11.7 | NC_000004 | 155484132 | 4265 | 35.02 | NC_000004 | 155484132 |
| LCN15 | **2.97** | **0.00** | 562 | 21 | NC_000009 | 139654086 | 2507 | 62.35 | NC_000009 | 139654086 |
| MCM3 | **2.96** | **0.02** | 495 | 7.63 | NC_000006 | 52128812 | 2830 | 22.58 | NC_000006 | 52128812 |
| SAMD4A | **2.95** | **0.04** | 4147 | 5.71 | NC_000014 | 55033815 | 8987 | 16.88 | NC_000014 | 55033815 |
| ROBO1 | **2.92** | **0.00** | 8025 | 13.58 | NC_000003 | 78646388 | 14158 | 39.62 | NC_000003 | 78646388 |
| AMBP | **2.91** | **0.00** | 2309 | 86.16 | NC_000009 | 116822407 | 12603 | 250.31 | NC_000009 | 116822407 |
| PRC1 | **2.91** | **0.03** | 622 | 6.84 | NC_000015 | 91509268 | 3298 | 19.91 | NC_000015 | 91509268 |
| GGH | **2.90** | **0.01** | 344 | 11.14 | NC_000008 | 63927638 | 1957 | 32.29 | NC_000008 | 63927638 |
| GANAB | **2.89** | **0.00** | 1231 | 16.88 | NC_000011 | 62392298 | 6785 | 48.8 | NC_000011 | 62392298 |
| FKBP9 | **2.86** | **0.03** | 615 | 7.33 | NC_000007 | 32997005 | 3178 | 20.96 | NC_000007 | 32997005 |
| FARP1 | **2.85** | **0.02** | 4063 | 8.53 | NC_000013 | 98794893 | 10085 | 24.34 | NC_000013 | 98794893 |
| SLC2A3 | **2.84** | **0.00** | 7102 | 94.78 | NC_000012 | 8071824 | 37534 | 269.16 | NC_000012 | 8071824 |
| DDX17 | **2.82** | **0.00** | 3332 | 25.77 | NC_000022 | 38879443 | 17943 | 72.74 | NC_000022 | 38879443 |
| HNF4A | **2.82** | **0.04** | 1108 | 6.48 | NC_000020 | 42984441 | 4569 | 18.3 | NC_000020 | 42984441 |
| HJURP | **2.81** | **0.00** | 810 | 13.23 | NC_000002 | 234745486 | 4945 | 37.14 | NC_000002 | 234745486 |
| PAH | **2.80** | **0.05** | 416 | 6.49E+00 | NC_000012 | 103232104 | 2840 | 18.13 | NC_000012 | 103232104 |
| CCDC14 | **2.79** | **0.01** | 1132 | 12.64 | NC_000003 | 123616152 | 6787 | 35.31 | NC_000003 | 123616152 |
| UBE2T | **2.79** | **0.04** | 148 | 7.23 | NC_000001 | 202300785 | 887 | 20.19 | NC_000001 | 202300785 |
| G6PC3 | **2.77** | **0.01** | 293 | 10 | NC_000017 | 42148098 | 1515 | 27.68 | NC_000017 | 42148098 |
| FANCI | **2.77** | **0.02** | 946 | 8.4 | NC_000015 | 89785634 | 5562 | 23.29 | NC_000015 | 89785634 |
| RNF43 | **2.77** | **0.03** | 1063 | 7.95E+00 | NC_000017 | 56431037 | 4478 | 2.20E+01 | NC_000017 | 56431037 |
| NFE2L3 | **2.77** | **0.03** | 650 | 7.45E+00 | NC_000007 | 26191847 | 2938 | 20.6 | NC_000007 | 26191847 |
| ORC6 | **2.75** | **0.04** | 275 | 7.46E+00 | NC_000016 | 46723558 | 1956 | 20.53 | NC_000016 | 46723558 |
| TMEM2 | **2.74** | **0.01** | 2801 | 12.64 | NC_000009 | 74298282 | 10187 | 34.59 | NC_000009 | 74298282 |
| CCNB1 | **2.72** | **0.00** | 707 | 1.48E+01 | NC_000005 | 68462837 | 3667 | 40.13 | NC_000005 | 68462837 |
| CLU | **2.71** | **0.03** | 439 | 8.62 | NC_000008 | 27454434 | 2277 | 23.32 | NC_000008 | 27454434 |
| SLC11A2 | **2.70** | **0.00** | 2409 | 15.63 | NC_000012 | 51373566 | 11336 | 42.18 | NC_000012 | 51373566 |
| SLC39A1 | **2.70** | **0.04** | 409 | 7.36 | NC_000001 | 153931575 | 2145 | 19.84 | NC_000001 | 153931575 |
| ITGAV | **2.69** | **0.01** | 2494 | 13.43 | NC_000002 | 187454058 | 10505 | 36.09 | NC_000002 | 187454058 |

| | | | | | | | | | | |
|---|---|---|---|---|---|---|---|---|---|---|
| MRFAP1L1 | **2.66** | **0.05** | 229 | 6.99 | NC_000004 | 6709428 | 1105 | 18.58 | NC_000004 | 6709428 |
| ZNF83 | **2.63** | **0.04** | 810 | 8.11 | NC_000019 | 53115617 | 3521 | 21.35 | NC_000019 | 53115617 |
| HMGN3 | **2.59** | **0.05** | 219 | 7.64 | NC_000006 | 79910962 | 1151 | 19.77 | NC_000006 | 79910962 |
| CASP8 | **2.58** | **0.01** | 1158 | 12.88 | NC_000002 | 202098166 | 5020 | 33.26 | NC_000002 | 202098166 |
| MPZL2 | **2.58** | **0.01** | 886 | 12.06 | NC_000011 | 118124131 | 4509 | 31.15 | NC_000011 | 118124131 |
| MALT1 | **2.56** | **0.00** | 3923 | 23.87 | NC_000018 | 56338618 | 13007 | 61.10 | NC_000018 | 56338618 |
| C2 | **2.56** | **0.02** | 674 | 11.09 | NC_000006 | 31865562 | 3200 | 28.44 | NC_000006 | 31865562 |
| GSN | **2.54** | **0.00** | 2076 | 27.68 | NC_000009 | 124030380 | 9316 | 70.19 | NC_000009 | 124030380 |
| ALDH18A1 | **2.54** | **0.00** | 1510 | 19.64 | NC_000010 | 97365686 | 7538 | 49.95 | NC_000010 | 97365686 |
| PIPOX | **2.54** | **0.04** | 478 | 8.85 | NC_000017 | 27369918 | 2375 | 22.45 | NC_000017 | 27369918 |
| APOA4 | **2.53** | **0.00** | 706 | 27.68 | NC_000011 | 116691418 | 3402 | 70.04 | NC_000011 | 116691418 |
| PLK1S1 | **2.53** | **0.00** | 2949 | 22.27 | NC_000020 | 21106624 | 19561 | 56.3 | NC_000020 | 21106624 |
| GRN | **2.52** | **0.00** | 857 | 17.76 | NC_000017 | 42422491 | 3913 | 44.8 | NC_000017 | 42422491 |
| LEPRE1 | **2.52** | **0.02** | 691 | 11.02 | NC_000001 | 43212006 | 3349 | 27.75 | NC_000001 | 43212006 |
| TPM1 | **2.52** | **0.02** | 1371 | 11.09 | NC_000015 | 63334838 | 7037 | 27.93 | NC_000015 | 63334838 |
| PPWD1 | **2.52** | **0.04** | 537 | 9 | NC_000005 | 64859063 | 2788 | 22.72 | NC_000005 | 64859063 |
| ALDH4A1 | **2.51** | **0.03** | 935 | 9.97 | NC_000001 | 19197924 | 4231 | 24.98 | NC_000001 | 19197924 |
| GLT8D1 | **2.50** | **0.04** | 533 | 9.84 | NC_000003 | 52728500 | 2527 | 24.65 | NC_000003 | 52728500 |
| CPE | **2.49** | **0.00** | 1499 | 26.2 | NC_000004 | 166300097 | 5726 | 65.13 | NC_000004 | 166300097 |
| ASAP2 | **2.49** | **0.03** | 2608 | 10.78 | NC_000002 | 9346894 | 7527 | 26.84 | NC_000002 | 9346894 |
| MSL1 | **2.46** | **0.04** | 1033 | 9.46 | NC_000017 | 38278235 | 4385 | 23.23 | NC_000017 | 38278235 |
| PLOD1 | **2.44** | **0.01** | 907 | 14.7 | NC_000001 | 11994724 | 4064 | 35.93 | NC_000001 | 11994724 |
| C3 | **2.42** | **0.00** | 13650 | 125.55 | NC_000019 | 6677846 | 54428 | 303.95 | NC_000019 | 6677846 |
| ITM2C | **2.42** | **0.02** | 558 | 14.37 | NC_000002 | 231729546 | 2534 | 34.72 | NC_000002 | 231729546 |
| SPINK1 | **2.42** | **0.04** | 245 | 10.38 | NC_000005 | 147204143 | 681 | 25.14 | NC_000005 | 147204143 |
| FANCL | **2.41** | **0.01** | 898 | 16.73 | NC_000002 | 58386378 | 4647 | 40.24 | NC_000002 | 58386378 |
| F10 | **2.41** | **0.04** | 526 | 10.16 | NC_000013 | 113777113 | 2311 | 24.49 | NC_000013 | 113777113 |
| PCED1A | **2.41** | **0.05** | 543 | 9.63 | NC_000020 | 2815960 | 3035 | 23.21 | NC_000020 | 2815960 |
| CRYZ | **2.40** | **0.01** | 798 | 16.88 | NC_000001 | 75171170 | 4010 | 40.49 | NC_000001 | 75171170 |
| PRKCSH | **2.39** | **0.01** | 1033 | 18.7 | NC_000019 | 11546111 | 4124 | 44.69 | NC_000019 | 11546111 |
| SRSF10 | **2.37** | **0.03** | 1200 | 11.96 | NC_000001 | 24292935 | 5695 | 28.39 | NC_000001 | 24292935 |
| HSD17B7 | **2.37** | **0.04** | 501 | 11.07 | NC_000001 | 162760496 | 2612 | 26.22 | NC_000001 | 162760496 |
| C16orf58 | **2.36** | **0.05** | 572 | 10.29 | NC_000016 | 31500796 | 2618 | 24.32 | NC_000016 | 31500796 |
| CD46 | **2.35** | **0.00** | 2625 | 37.57 | NC_000001 | 207925383 | 12433 | 88.37 | NC_000001 | 207925383 |
| RRM2 | **2.35** | **0.02** | 895 | 13.85 | NC_000002 | 10262695 | 3942 | 32.59 | NC_000002 | 10262695 |
| ALDH1A1 | **2.34** | **0.01** | 1163 | 21.17 | NC_000009 | 75515578 | 5150 | 49.57 | NC_000009 | 75515578 |
| CFI | **2.33** | **0.00** | 1923 | 38.67 | NC_000004 | 110661848 | 9190 | 89.94 | NC_000004 | 110661848 |
| HSPA12A | **2.33** | **0.01** | 2649 | 20.03 | NC_000010 | 118430703 | 11745 | 46.63 | NC_000010 | 118430703 |
| TXNDC5 | **2.32** | **0.01** | 1351 | 17.69 | NC_000006 | 7881483 | 5320 | 41.07 | NC_000006 | 7881483 |
| SLC37A4 | **2.32** | **0.04** | 588 | 11.59 | NC_000011 | 118895061 | 2871 | 26.87 | NC_000011 | 118895061 |
| SERPINA6 | **2.30** | **0.01** | 644 | 19.68 | NC_000014 | 94770585 | 3001 | 45.2 | NC_000014 | 94770585 |
| DHCR24 | **2.30** | **0.05** | 1018 | 11.62 | NC_000001 | 55315300 | 4102 | 26.73 | NC_000001 | 55315300 |
| TIA1 | **2.28** | **0.01** | 2666 | 20.47 | NC_000002 | 70436576 | 11834 | 46.72 | NC_000002 | 70436576 |
| C4A | **2.28** | **0.04** | 1255 | 12.16 | NC_000006 | 31949834 | 5230 | 27.78 | NC_000006 | 31949834 |
| DPP4 | **2.28** | **0.04** | 1419 | 12.28 | NC_000002 | 162848751 | 4498 | 28.05 | NC_000002 | 162848751 |
| RFC4 | **2.28** | **0.04** | 386 | 12.85 | NC_000003 | 186507681 | 1923 | 29.25 | NC_000003 | 186507681 |
| ALPK2 | **2.26** | **0.00** | 5934 | 28.61 | NC_000018 | 56148479 | 25248 | 64.59 | NC_000018 | 56148479 |
| FGG | **2.25** | **0.00** | 2594 | 73.99 | NC_000004 | 155525286 | 11621 | 166.28 | NC_000004 | 155525286 |
| KRT19 | **2.25** | **0.00** | 846 | 28.77 | NC_000017 | 39679869 | 3520 | 64.6 | NC_000017 | 39679869 |
| APOB | **2.24** | **0.00** | 6992 | 25.68 | NC_000002 | 21224301 | 27483 | 57.42 | NC_000002 | 21224301 |
| IGF2 | **2.22** | **0.00** | 10752 | 93.97 | NC_000011 | 2150346 | 45576 | 208.54 | NC_000011 | 2150346 |
| LAMB1 | **2.22** | **0.00** | 4313 | 31.35 | NC_000007 | 107564244 | 16893 | 69.59 | NC_000007 | 107564244 |
| ARL6IP1 | **2.22** | **0.01** | 1063 | 23.38 | NC_000016 | 18802991 | 4478 | 51.8 | NC_000016 | 18802991 |
| MOGS | **2.22** | **0.02** | 909 | 17.42 | NC_000002 | 74688184 | 3831 | 38.73 | NC_000002 | 74688184 |
| NUP107 | **2.22** | **0.02** | 1282 | 18.7 | NC_000012 | 69080731 | 5561 | 41.53 | NC_000012 | 69080731 |
| NOMO1 | **2.22** | **0.03** | 1762 | 15.21 | NC_000016 | 14927643 | 5700 | 33.69 | NC_000016 | 14927643 |
| TMEM98 | **2.22** | **0.05** | 447 | 13.09 | NC_000017 | 31254928 | 1938 | 29.05 | NC_000017 | 31254928 |
| BMP4 | **2.21** | **0.01** | 889 | 20.24 | NC_000014 | 54416454 | 3906 | 44.79 | NC_000014 | 54416454 |
| PRDX4 | **2.21** | **0.02** | 603 | 18.54 | NC_000023 | 23682377 | 2247 | 41.03 | NC_000023 | 23682377 |
| AURKB | **2.21** | **0.05** | 321 | 12.48 | NC_000017 | 8108049 | 1478 | 27.55 | NC_000017 | 8108049 |
| SUMF2 | **2.20** | **0.00** | 1609 | 27.12 | NC_000007 | 56131700 | 6277 | 59.67 | NC_000007 | 56131700 |
| IL17RB | **2.18** | **0.00** | 3197 | 60.66 | NC_000003 | 53880577 | 14241 | 131.97 | NC_000003 | 53880577 |
| TBC1D8 | **2.18** | **0.01** | 3902 | 25.06 | NC_000002 | 101623690 | 11295 | 54.55 | NC_000002 | 101623690 |
| ASAH1 | **2.18** | **0.03** | 1244 | 16.36 | NC_000008 | 17913925 | 4562 | 35.63 | NC_000008 | 17913925 |
| ERBB3 | **2.17** | **0.05** | 1554 | 13.41 | NC_000012 | 56473809 | 6224 | 29.05 | NC_000012 | 56473809 |
| SMYD2 | **2.16** | **0.02** | 1158 | 20.4 | NC_000001 | 214454565 | 4872 | 43.99 | NC_000001 | 214454565 |
| DYNLT1 | **2.15** | **0.03** | 318 | 16.38 | NC_000006 | 159057506 | 1236 | 35.23 | NC_000006 | 159057506 |
| TGFBI | **2.14** | **0.00** | 2835 | 51.91 | NC_000005 | 135364584 | 11387 | 111.31 | NC_000005 | 135364584 |

| | | | | | | | | | | |
|---|---|---|---|---|---|---|---|---|---|---|
| F2 | **2.14** | **0.00** | 1918 | 43.08 | NC_000011 | 46740743 | 7440 | 92.04 | NC_000011 | 46740743 |
| CHDH | **2.13** | **0.03** | 1180 | 17.17 | NC_000003 | 53850324 | 5342 | 36.5 | NC_000003 | 53850324 |
| FBXO17 | **2.13** | **0.05** | 1484 | 14.35 | NC_000019 | 39432041 | 4577 | 30.61 | NC_000019 | 39432041 |
| APOE | **2.11** | **0.00** | 755 | 31.47 | NC_000019 | 45409039 | 2865 | 66.31 | NC_000019 | 45409039 |
| AGT | **2.10** | **0.00** | 3197 | 69.47 | NC_000001 | 230838269 | 13689 | 146.2 | NC_000001 | 230838269 |
| MT1E | **2.10** | **0.00** | 782 | 47.32 | NC_000016 | 56659557 | 3245 | 99.36 | NC_000016 | 56659557 |
| FAM156A | **2.10** | **0.02** | 1352 | 20.74 | NC_000023 | 52976462 | 5970 | 43.46 | NC_000023 | 52976462 |
| ENC1 | **2.10** | **0.05** | 1622 | 15.18 | NC_000005 | 73923231 | 6750 | 31.81 | NC_000005 | 73923231 |
| GPX2 | **2.09** | **0.00** | 1349 | 67.95 | NC_000014 | 65405870 | 5732 | 142.05 | NC_000014 | 65405870 |
| AFMID | **2.06** | **0.02** | 1230 | 24.61 | NC_000017 | 76183398 | 5055 | 50.78 | NC_000017 | 76183398 |
| CNOT11 | **2.05** | **0.02** | 1271 | 24.08 | NC_000002 | 101869345 | 5644 | 49.47 | NC_000002 | 101869345 |
| PRDX3 | **2.05** | **0.03** | 637 | 19.3 | NC_000010 | 120927215 | 2956 | 39.65 | NC_000010 | 120927215 |
| TGOLN2 | **2.05** | **0.03** | 2462 | 21.72 | NC_000002 | 85545141 | 9666 | 44.51 | NC_000002 | 85545141 |
| SERPINA5 | **2.04** | **0.00** | 1905 | 42.03 | NC_000014 | 95047706 | 6988 | 85.78 | NC_000014 | 95047706 |
| TMEM37 | **2.03** | **0.03** | 753 | 21.49 | NC_000002 | 120187501 | 2855 | 43.59 | NC_000002 | 120187501 |
| ATRAID | **2.03** | **0.05** | 465 | 17.75 | NC_000002 | 27434899 | 1812 | 36.06 | NC_000002 | 27434899 |
| RBP4 | **2.02** | **0.00** | 1210 | 56.41 | NC_000010 | 95351593 | 4477 | 114.09 | NC_000010 | 95351593 |
| GSTK1 | **2.02** | **0.02** | 572 | 23.97 | NC_000007 | 142960506 | 2346 | 48.31 | NC_000007 | 142960506 |
| TMEM141 | **2.01** | **0.03** | 393 | 23.2 | NC_000009 | 139685777 | 1509 | 46.54 | NC_000009 | 139685777 |
| CTDSP1 | **2.01** | **0.04** | 1011 | 19.82 | NC_000002 | 219263061 | 3978 | 39.93 | NC_000002 | 219263061 |
| TUBB | **2.00** | **0.00** | 2391 | 46.09 | NC_000006 | 30688012 | 9112 | 92.24 | NC_000006 | 30688012 |
| CNPY2 | **1.99** | **0.04** | 807 | 20.45 | NC_000012 | 56704212 | 2801 | 40.74 | NC_000012 | 56704212 |
| ARGLU1 | **1.98** | **0.00** | 1946 | 45.08 | NC_000013 | 107195662 | 9376 | 89.44 | NC_000013 | 107195662 |
| ANXA4 | **1.97** | **0.00** | 5979 | 76.28 | NC_000002 | 69947946 | 19271 | 150.59 | NC_000002 | 69947946 |
| COX6A1 | **1.96** | **0.00** | 1213 | 105.59 | NC_000012 | 120875893 | 4015 | 206.79 | NC_000012 | 120875893 |
| VDAC3 | **1.96** | **0.02** | 1038 | 28.23 | NC_000008 | 42249279 | 4734 | 55.46 | NC_000008 | 42249279 |
| SMAD3 | **1.95** | **0.04** | 5217 | 22.64 | NC_000015 | 67358036 | 13493 | 44.09 | NC_000015 | 67358036 |
| HNRNPA2B1 | **1.94** | **0.00** | 6641 | 96 | NC_000007 | 26229547 | 24553 | 185.78 | NC_000007 | 26229547 |
| ANKRD1 | **1.94** | **0.03** | 1261 | 26.75 | NC_000010 | 92671857 | 5121 | 51.91 | NC_000010 | 92671857 |
| CA9 | **1.92** | **0.01** | 1358 | 40 | NC_000009 | 35673915 | 4869 | 76.91 | NC_000009 | 35673915 |
| APOA1 | **1.91** | **0.00** | 5480 | 321.9 | NC_000011 | 116706467 | 19924 | 614.82 | NC_000011 | 116706467 |
| SLC2A1 | **1.91** | **0.00** | 9116 | 120.87 | NC_000001 | 43391046 | 29898 | 231.28 | NC_000001 | 43391046 |
| MAT2A | **1.90** | **0.01** | 2641 | 46.43 | NC_000002 | 85766101 | 10345 | 88.22 | NC_000002 | 85766101 |
| EPCAM | **1.89** | **0.00** | 2081 | 54.05 | NC_000002 | 47596287 | 6711 | 102.35 | NC_000002 | 47596287 |
| TMEM59 | **1.88** | **0.04** | 1199 | 25.82 | NC_000001 | 54497346 | 3680 | 48.61 | NC_000001 | 54497346 |
| GTF2I | **1.88** | **0.05** | 2992 | 23.9 | NC_000007 | 74071991 | 10317 | 44.98 | NC_000007 | 74071991 |
| TM4SF1 | **1.87** | **0.00** | 7456 | 212.22 | NC_000003 | 149086805 | 24984 | 397.06 | NC_000003 | 149086805 |
| SEPP1 | **1.87** | **0.03** | 1407 | 32.62 | NC_000005 | 42799982 | 4841 | 60.86 | NC_000005 | 42799982 |
| ACAA1 | **1.87** | **0.04** | 1017 | 26.56 | NC_000003 | 38164201 | 3622 | 49.6 | NC_000003 | 38164201 |
| HGD | **1.87** | **0.05** | 1952 | 24.65 | NC_000003 | 120347015 | 11283 | 46.16 | NC_000003 | 120347015 |
| DDOST | **1.86** | **0.01** | 2115 | 41.27 | NC_000001 | 20978260 | 5772 | 76.63 | NC_000001 | 20978260 |
| VPS25 | **1.86** | **0.04** | 654 | 28.47 | NC_000017 | 40925454 | 2399 | 52.97 | NC_000017 | 40925454 |
| BSG | **1.85** | **0.00** | 2989 | 66.89 | NC_000019 | 571277 | 9961 | 123.9 | NC_000019 | 571277 |
| SLC3A2 | **1.84** | **0.00** | 3376 | 60.05 | NC_000011 | 62623484 | 10214 | 110.71 | NC_000011 | 62623484 |
| B2M | **1.83** | **0.00** | 2036 | 88.98 | NC_000015 | 45003685 | 5933 | 162.48 | NC_000015 | 45003685 |
| H2AFZ | **1.83** | **0.01** | 996 | 44.02 | NC_000004 | 100869242 | 3448 | 80.38 | NC_000004 | 100869242 |
| SERPINA1 | **1.82** | **0.00** | 26561 | 406.51 | NC_000014 | 94843084 | 92233 | 737.87 | NC_000014 | 94843084 |
| CPNE1 | **1.82** | **0.01** | 3229 | 45.75 | NC_000020 | 34213953 | 10945 | 83.45 | NC_000020 | 34213953 |
| FSTL3 | **1.81** | **0.00** | 4718 | 105.7 | NC_000019 | 676389 | 16411 | 191.66 | NC_000019 | 676389 |
| SERPINF1 | **1.80** | **0.00** | 1906 | 63.06 | NC_000017 | 1665259 | 6473 | 113.56 | NC_000017 | 1665259 |
| APOC3 | **1.80** | **0.01** | 509 | 53.79 | NC_000011 | 116700624 | 1817 | 96.86 | NC_000011 | 116700624 |
| CALM2 | **1.79** | **0.00** | 3067 | 77.29 | NC_000002 | 47387221 | 10033 | 138.51 | NC_000002 | 47387221 |
| APLP2 | **1.78** | **0.01** | 4365 | 54.83 | NC_000011 | 129939716 | 13812 | 97.72 | NC_000011 | 129939716 |
| TMED9 | **1.75** | **0.02** | 1222 | 49.4 | NC_000005 | 177019213 | 4034 | 86.44 | NC_000005 | 177019213 |
| CIRBP | **1.73** | **0.00** | 3906 | 113.58 | NC_000019 | 1269267 | 13805 | 196.95 | NC_000019 | 1269267 |
| ID1 | **1.73** | **0.02** | 1100 | 51.36 | NC_000020 | 30193086 | 3615 | 88.67 | NC_000020 | 30193086 |
| CTSD | **1.73** | **0.05** | 1441 | 36.83 | NC_000011 | 1773982 | 4578 | 63.59 | NC_000011 | 1773982 |
| CST3 | **1.71** | **0.01** | 2892 | 65.9 | NC_000020 | 23608534 | 9777 | 112.74 | NC_000020 | 23608534 |
| DDX5 | **1.71** | **0.01** | 5498 | 62.48 | NC_000017 | 62494374 | 21127 | 106.88 | NC_000017 | 62494374 |
| AHSA2 | **1.71** | **0.05** | 3336 | 38.06 | NC_000002 | 61404553 | 10578 | 65.15 | NC_000002 | 61404553 |
| CANX | **1.70** | **0.05** | 4608 | 40.9 | NC_000005 | 179125019 | 13274 | 69.5 | NC_000005 | 179125019 |
| PON2 | **1.66** | **0.01** | 2811 | 76.94 | NC_000007 | 95034174 | 10892 | 127.54 | NC_000007 | 95034174 |
| PDIA3 | **1.63** | **0.03** | 4487 | 63.17 | NC_000015 | 44038326 | 12334 | 102.76 | NC_000015 | 44038326 |
| MT1X | **1.62** | **0.00** | 1086 | 116 | NC_000016 | 56716382 | 3581 | 188.13 | NC_000016 | 56716382 |
| ABCC2 | **1.62** | **0.02** | 9705 | 79.35 | NC_000010 | 101542457 | 24822 | 128.79 | NC_000010 | 101542457 |
| TUBA1B | **1.60** | **0.02** | 2653 | 77.07 | NC_000012 | 49521565 | 8116 | 123.41 | NC_000012 | 49521565 |
| PABPC1 | **1.60** | **0.02** | 5277 | 86.07 | NC_000008 | 101715144 | 15756 | 137.74 | NC_000008 | 101715144 |
| CFB | **1.58** | **0.04** | 3264 | 66.67 | NC_000006 | 31913721 | 10034 | 105.4 | NC_000006 | 31913721 |

| | | | | | | | | | | | |
|---|---|---|---|---|---|---|---|---|---|---|---|
| NME1-NME2 | **1.55** | **0.05** | 1651 | 73 | NC_000017 | 49230897 | 4522 | 112.82 | NC_000017 | 49230897 |
| PKM | **1.53** | **0.04** | 6731 | 89.57 | NC_000015 | 72491370 | 21886 | 137.45 | NC_000015 | 72491370 |
| SERPINE1 | **1.51** | **0.00** | 54688 | 414.29 | NC_000007 | 100770370 | 85266 | 624.79 | NC_000007 | 100770370 |
| ORM1 | **1.35** | **0.00** | 8816 | 573.75 | NC_000009 | 117085303 | 22074 | 776.51 | NC_000009 | 117085303 |
| RPS21 | **1.32** | **0.00** | 9135 | 1,228.96 | NC_000020 | 60962121 | 21647 | 1,616.16 | NC_000020 | 60962121 |
| RPL37A | **1.04** | **0.02** | 9640 | 1,111.58 | NC_000002 | 217363520 | 17491 | 1,158.72 | NC_000002 | 217363520 |
| RPL41 | **1.03** | **0.02** | 9983 | 877.7 | NC_000012 | 56510374 | 19004 | 907.28 | NC_000012 | 56510374 |
| APOA2 | **1.02** | **0.00** | 12777 | 1,485.47 | NC_000001 | 161192083 | 24482 | 1,515.99 | NC_000001 | 161192083 |
| RPL5 | **-1.01** | **0.03** | 8637 | 391.01 | NC_000001 | 93297594 | 15560 | 387.92 | NC_000001 | 93297594 |
| RPL27 | **-1.01** | **0.03** | 3672 | 389.87 | NC_000017 | 41150446 | 6753 | 386.03 | NC_000017 | 41150446 |
| RPS27A | **-1.02** | **0.04** | 8370 | 319.07 | NC_000002 | 55459039 | 14778 | 312.42 | NC_000002 | 55459039 |
| RPS18 | **-1.03** | **0.00** | 11471 | 1,122.58 | NC_000006 | 33239852 | 20602 | 1,086.95 | NC_000006 | 33239852 |
| RPS11 | **-1.04** | **0.00** | 6321 | 477.88 | NC_000019 | 49999622 | 10606 | 457.5 | NC_000019 | 49999622 |
| ACTG1 | **-1.04** | **0.02** | 11770 | 319.68 | NC_000017 | 79476997 | 21455 | 306.98 | NC_000017 | 79476997 |
| RPS13 | **-1.04** | **0.04** | 3260 | 259.79 | NC_000011 | 17095939 | 5029 | 249.44 | NC_000011 | 17095939 |
| PTTG1IP | **-1.04** | **0.05** | 17961 | 239.7 | NC_000021 | 46269500 | 35822 | 230.38 | NC_000021 | 46269500 |
| RPS8 | **-1.05** | **0.01** | 5085 | 328.2 | NC_000001 | 45241213 | 8454 | 311.5 | NC_000001 | 45241213 |
| RPL13A | **-1.06** | **0.00** | 10734 | 498.17 | NC_000019 | 49990811 | 18633 | 467.8 | NC_000019 | 49990811 |
| RPL35A | **-1.06** | **0.02** | 3368 | 276.76 | NC_000003 | 197677023 | 5550 | 261.61 | NC_000003 | 197677023 |
| RPS20 | **-1.07** | **0.04** | 5385 | 206.93 | NC_000008 | 56980739 | 8912 | 193.97 | NC_000008 | 56980739 |
| RPS4Y1 | **-1.07** | **0.05** | 5861 | 191.38 | NC_000024 | 2709623 | 11872 | 179.45 | NC_000024 | 2709623 |
| RPL14 | **-1.08** | **0.02** | 4097 | 226.55 | NC_000003 | 40498783 | 6857 | 210.21 | NC_000003 | 40498783 |
| HSP90AB1 | **-1.08** | **0.05** | 8726 | 161.51 | NC_000006 | 44213903 | 15134 | 149.47 | NC_000006 | 44213903 |
| LGALS3 | **-1.08** | **0.05** | 3442 | 156.83 | NC_000014 | 55595935 | 7006 | 144.9 | NC_000014 | 55595935 |
| RPL12 | **-1.09** | **0.00** | 6267 | 406.64 | NC_000009 | 130209953 | 10711 | 371.86 | NC_000009 | 130209953 |
| RPS25 | **-1.09** | **0.00** | 4948 | 425.37 | NC_000011 | 118886422 | 7427 | 391.64 | NC_000011 | 118886422 |
| NOP56 | **-1.09** | **0.04** | 7743 | 172.38 | NC_000020 | 2633178 | 13052 | 158.42 | NC_000020 | 2633178 |
| MYL6 | **-1.09** | **0.05** | 4185 | 161.34 | NC_000012 | 56552045 | 6665 | 148.3 | NC_000012 | 56552045 |
| TPT1 | **-1.10** | **0.00** | 11401 | 383.99 | NC_000013 | 45911005 | 18282 | 349.69 | NC_000013 | 45911005 |
| RPS9 | **-1.10** | **0.01** | 4458 | 260.53 | NC_000019 | 54704608 | 7032 | 236.45 | NC_000019 | 54704608 |
| RPS12 | **-1.11** | **0.00** | 6274 | 616.69 | NC_000006 | 133135708 | 10399 | 554.61 | NC_000006 | 133135708 |
| RPL39 | **-1.11** | **0.00** | 3759 | 496.73 | NC_000023 | 118920467 | 6377 | 446.45 | NC_000023 | 118920467 |
| RPS16 | **-1.11** | **0.00** | 5535 | 322.49 | NC_000019 | 39923847 | 8989 | 289.9 | NC_000019 | 39923847 |
| RPS15 | **-1.11** | **0.05** | 2023 | 138.68 | NC_000019 | 1438363 | 3091 | 125.39 | NC_000019 | 1438363 |
| TMSB10 | **-1.12** | **0.00** | 7673 | 908.16 | NC_000002 | 85132763 | 13016 | 812.14 | NC_000002 | 85132763 |
| NPM1 | **-1.12** | **0.01** | 7427 | 197.74 | NC_000005 | 170814652 | 11860 | 177.07 | NC_000005 | 170814652 |
| CHCHD2 | **-1.12** | **0.03** | 2611 | 146.58 | NC_000007 | 56169266 | 4062 | 130.89 | NC_000007 | 56169266 |
| TRA2A | **-1.13** | **0.05** | 5808 | 109.89 | NC_000007 | 23544399 | 11590 | 97.08 | NC_000007 | 23544399 |
| RPL7 | **-1.15** | **0.00** | 6896 | 443.87 | NC_000008 | 74202874 | 11143 | 387.65 | NC_000008 | 74202874 |
| RPL18A | **-1.15** | **0.02** | 2162 | 136.68 | NC_000019 | 17970687 | 2957 | 118.54 | NC_000019 | 17970687 |
| RPL38 | **-1.17** | **0.00** | 3649 | 464.11 | NC_000017 | 72199795 | 5531 | 395.64 | NC_000017 | 72199795 |
| RPS14 | **-1.17** | **0.01** | 7261 | 166.79 | NC_000005 | 149822712 | 10139 | 142.46 | NC_000005 | 149822712 |
| ASGR1 | **-1.17** | **0.01** | 6201 | 150.56 | NC_000017 | 7076750 | 9463 | 128.48 | NC_000017 | 7076750 |
| RPL18 | **-1.17** | **0.01** | 2796 | 139.42 | NC_000019 | 49118584 | 3907 | 118.66 | NC_000019 | 49118584 |
| EIF6 | **-1.17** | **0.02** | 3803 | 133.41 | NC_000020 | 33866709 | 6162 | 114.2 | NC_000020 | 33866709 |
| RPL35 | **-1.18** | **0.00** | 3418 | 314.9 | NC_000009 | 127620158 | 4780 | 266.94 | NC_000009 | 127620158 |
| RPL17 | **-1.18** | **0.00** | 5816 | 224.6 | NC_000018 | 47014851 | 8503 | 1.91E+02 | NC_000018 | 47014851 |
| FAU | **-1.18** | **0.01** | 1760 | 152.09 | NC_000011 | 64888099 | 2574 | 128.39 | NC_000011 | 64888099 |
| ANXA5 | **-1.18** | **0.02** | 4942 | 124.33 | NC_000004 | 122589152 | 7646 | 105.54 | NC_000004 | 122589152 |
| YBX1 | **-1.18** | **0.03** | 4008 | 110.89 | NC_000001 | 43148060 | 6363 | 94.27 | NC_000001 | 43148060 |
| ALDOA | **-1.19** | **0.00** | 12535 | 186.96 | NC_000016 | 30064411 | 19196 | 157.26 | NC_000016 | 30064411 |
| RPS3A | **-1.20** | **0.00** | 9673 | 264.7 | NC_000004 | 152020725 | 15153 | 221.28 | NC_000004 | 152020725 |
| DDIT4 | **-1.20** | **0.02** | 3459 | 112.45 | NC_000010 | 74033677 | 5509 | 93.93 | NC_000010 | 74033677 |
| AUP1 | **-1.20** | **0.02** | 3879 | 114.46 | NC_000002 | 74753775 | 5964 | 95.5 | NC_000002 | 74753775 |
| SEC61B | **-1.20** | **0.04** | 1240 | 84.41 | NC_000009 | 101984570 | 2111 | 70.55 | NC_000009 | 101984570 |
| RPL4 | **-1.21** | **0.00** | 10423 | 290.24 | NC_000015 | 66791653 | 15864 | 239.8 | NC_000015 | 66791653 |
| RPL10A | **-1.21** | **0.00** | 5442 | 225.56 | NC_000006 | 35436178 | 7863 | 186.24 | NC_000006 | 35436178 |
| SRSF2 | **-1.21** | **0.01** | 7689 | 149.4 | NC_000017 | 74730197 | 12066 | 123.16 | NC_000017 | 74730197 |
| RPS19 | **-1.22** | **0.00** | 7099 | 404.63 | NC_000019 | 42363988 | 10601 | 330.82 | NC_000019 | 42363988 |
| RPL8 | **-1.22** | **0.00** | 4634 | 250.37 | NC_000008 | 146015152 | 6975 | 205.74 | NC_000008 | 146015152 |
| H3F3B | **-1.23** | **0.01** | 5747 | 121.6 | NC_000017 | 73772515 | 8821 | 98.46 | NC_000017 | 73772515 |
| ATP5E | **-1.24** | **0.00** | 2321 | 284.33 | NC_000020 | 57603733 | 3499 | 229.62 | NC_000020 | 57603733 |
| HNRNPH1 | **-1.25** | **0.00** | 14722 | 302.68 | NC_000005 | 179041179 | 25277 | 241.37 | NC_000005 | 179041179 |
| MST1 | **-1.25** | **0.00** | 7323 | 170.08 | NC_000003 | 49721380 | 11256 | 136.44 | NC_000003 | 49721380 |
| PSMB1 | **-1.25** | **0.01** | 2373 | 104.54 | NC_000006 | 170844204 | 4113 | 83.51 | NC_000006 | 170844204 |
| RBM39 | **-1.26** | **0.00** | 34747 | 499.85 | NC_000020 | 34291531 | 52695 | 397.21 | NC_000020 | 34291531 |
| PFDN5 | **-1.26** | **0.00** | 3041 | 180.77 | NC_000012 | 53689235 | 4819 | 143.17 | NC_000012 | 53689235 |
| RPL34 | **-1.27** | **0.00** | 4598 | 205.81 | NC_000004 | 109541722 | 6243 | 161.71 | NC_000004 | 109541722 |

| | | | | | | | | | | |
|---|---|---|---|---|---|---|---|---|---|---|
| EEF2 | **-1.27** | **0.00** | 11003 | 183.33 | NC_000019 | 3976054 | 15814 | 144.55 | NC_000019 | 3976054 |
| GDI2 | **-1.27** | **0.04** | 3236 | 61.2 | NC_000010 | 5807186 | 4822 | 48.05 | NC_000010 | 5807186 |
| OAZ1 | **-1.28** | **0.00** | 3465 | 143.38 | NC_000019 | 2269520 | 4868 | 112.24 | NC_000019 | 2269520 |
| RABGGTB | **-1.28** | **0.02** | 2961 | 81.07 | NC_000001 | 76251879 | 5231 | 63.58 | NC_000001 | 76251879 |
| IER3 | **-1.28** | **0.05** | 1273 | 58.83 | NC_000006 | 30710976 | 1898 | 46 | NC_000006 | 30710976 |
| RPS6 | **-1.29** | **0.00** | 10040 | 376.3 | NC_000009 | 19376253 | 13757 | 291.61 | NC_000009 | 19376253 |
| CYB5A | **-1.29** | **0.03** | 2343 | 68.19 | NC_000018 | 71920527 | 3387 | 52.91 | NC_000018 | 71920527 |
| CIB1 | **-1.29** | **0.04** | 1658 | 58.31 | NC_000015 | 90773477 | 2486 | 45.16 | NC_000015 | 90773477 |
| NDUFV2 | **-1.30** | **0.00** | 2949 | 108.19 | NC_000018 | 9102628 | 4958 | 83.1 | NC_000018 | 9102628 |
| NOP58 | **-1.30** | **0.01** | 4623 | 95.43 | NC_000002 | 203130515 | 7414 | 73.68 | NC_000002 | 203130515 |
| RAC1 | **-1.30** | **0.02** | 5658 | 72.44 | NC_000007 | 6414126 | 5860 | 55.7 | NC_000007 | 6414126 |
| EIF4G2 | **-1.30** | **0.02** | 6115 | 70.44 | NC_000011 | 10818593 | 8798 | 54.16 | NC_000011 | 10818593 |
| EIF3B | **-1.31** | **0.02** | 5046 | 66.93 | NC_000007 | 2394474 | 6905 | 51.02 | NC_000007 | 2394474 |
| CCT8 | **-1.31** | **0.02** | 3036 | 70.65 | NC_000021 | 30428647 | 4498 | 53.77 | NC_000021 | 30428647 |
| EIF3D | **-1.31** | **0.03** | 2751 | 66.41 | NC_000022 | 36906897 | 4034 | 50.74 | NC_000022 | 36906897 |
| RPL36 | **-1.32** | **0.00** | 2615 | 206.65 | NC_000019 | 5690272 | 3381 | 156.58 | NC_000019 | 5690272 |
| NCL | **-1.32** | **0.00** | 7035 | 129.72 | NC_000002 | 232319459 | 9586 | 98.08 | NC_000002 | 232319459 |
| ADM | **-1.32** | **0.00** | 3436 | 128.93 | NC_000011 | 10326642 | 4869 | 97.47 | NC_000011 | 10326642 |
| RPLP2 | **-1.32** | **0.01** | 3849 | 84.42 | NC_000011 | 808841 | 5381 | 64.08 | NC_000011 | 808841 |
| GNL3 | **-1.32** | **0.02** | 3142 | 74.91 | NC_000003 | 52719936 | 4780 | 56.93 | NC_000003 | 52719936 |
| RPL27A | **-1.32** | **0.03** | 5977 | 59.25 | NC_000011 | 8703995 | 8012 | 45.04 | NC_000011 | 8703995 |
| RPLP1 | **-1.34** | **0.00** | 4658 | 467 | NC_000015 | 69745159 | 6529 | 349.76 | NC_000015 | 69745159 |
| TPD52L2 | **-1.34** | **0.01** | 4128 | 78.39 | NC_000020 | 62496581 | 6948 | 58.4 | NC_000020 | 62496581 |
| EIF3I | **-1.34** | **0.03** | 1693 | 56.74 | NC_000001 | 32687971 | 2228 | 42.19 | NC_000001 | 32687971 |
| SLC25A3 | **-1.35** | **0.00** | 5605 | 128.4 | NC_000012 | 98987403 | 7855 | 94.88 | NC_000012 | 98987403 |
| PSMB4 | **-1.35** | **0.00** | 2719 | 115.96 | NC_000001 | 151372041 | 3349 | 86.02 | NC_000001 | 151372041 |
| LAMTOR5 | **-1.35** | **0.03** | 1275 | 56.76 | NC_000001 | 110943875 | 1669 | 41.92 | NC_000001 | 110943875 |
| ROMO1 | **-1.36** | **0.01** | 804 | 76.43 | NC_000020 | 34287232 | 1031 | 56.03 | NC_000020 | 34287232 |
| PSMD7 | **-1.36** | **0.05** | 1674 | 44.82 | NC_000016 | 74330673 | 2373 | 32.91 | NC_000016 | 74330673 |
| BHLHE40 | **-1.37** | **0.00** | 8491 | 142.94 | NC_000003 | 5021097 | 11529 | 104 | NC_000003 | 5021097 |
| LCN2 | **-1.37** | **0.01** | 1371 | 74.03 | NC_000009 | 130911732 | 1754 | 53.9 | NC_000009 | 130911732 |
| NRBP1 | **-1.37** | **0.05** | 2086 | 42.91 | NC_000002 | 27651473 | 2815 | 31.23 | NC_000002 | 27651473 |
| RPL3 | **-1.38** | **0.00** | 17528 | 678.88 | NC_000022 | 39708887 | 22933 | 491.21 | NC_000022 | 39708887 |
| HSPA5 | **-1.38** | **0.00** | 17836 | 248.48 | NC_000009 | 127997127 | 24339 | 1.80E+02 | NC_000009 | 127997127 |
| ATF4 | **-1.38** | **0.00** | 6149 | 174.46 | NC_000022 | 39916569 | 8448 | 126.09 | NC_000022 | 39916569 |
| EDF1 | **-1.38** | **0.00** | 1774 | 86.39 | NC_000009 | 139756566 | 2368 | 62.78 | NC_000009 | 139756566 |
| RPL24 | **-1.39** | **0.00** | 4116 | 377.14 | NC_000003 | 101399934 | 5401 | 271.34 | NC_000003 | 101399934 |
| HSPD1 | **-1.39** | **0.00** | 12594 | 235.88 | NC_000002 | 198351308 | 17986 | 169.94 | NC_000002 | 198351308 |
| RPL9 | **-1.40** | **0.00** | 6358 | 362.5 | NC_000004 | 39455744 | 8555 | 258.34 | NC_000004 | 39455744 |
| IL32 | **-1.40** | **0.00** | 7276 | 265.85 | NC_000016 | 3115313 | 9037 | 189.37 | NC_000016 | 3115313 |
| SLC51A | **-1.40** | **0.00** | 3708 | 108.02 | NC_000003 | 195943383 | 5650 | 76.93 | NC_000003 | 195943383 |
| SEMA3B | **-1.40** | **0.01** | 4099 | 63.42 | NC_000003 | 50305040 | 5553 | 45.22 | NC_000003 | 50305040 |
| ILF2 | **-1.40** | **0.02** | 2102 | 54.04 | NC_000001 | 153634264 | 2775 | 38.61 | NC_000001 | 153634264 |
| DSTN | **-1.40** | **0.03** | 2547 | 47.23 | NC_000020 | 17549818 | 3040 | 33.64 | NC_000020 | 17549818 |
| RPL36AL | **-1.41** | **0.00** | 1139 | 109.16 | NC_000014 | 50085406 | 1503 | 77.21 | NC_000014 | 50085406 |
| C8orf4 | **-1.41** | **0.02** | 1826 | 57.1 | NC_000008 | 40010987 | 2470 | 40.58 | NC_000008 | 40010987 |
| EIF4A1 | **-1.42** | **0.00** | 9281 | 228.64 | NC_000017 | 7476024 | 11135 | 160.79 | NC_000017 | 7476024 |
| S100P | **-1.42** | **0.00** | 1941 | 203.43 | NC_000004 | 6695566 | 2829 | 142.8 | NC_000004 | 6695566 |
| RAB24 | **-1.42** | **0.02** | 1579 | 48.82 | NC_000005 | 176728199 | 2128 | 34.37 | NC_000005 | 176728199 |
| JAG1 | **-1.43** | **0.05** | 5411 | 36.35 | NC_000020 | 10618332 | 5731 | 25.48 | NC_000020 | 10618332 |
| GDF15 | **-1.44** | **0.00** | 2696 | 105.41 | NC_000019 | 18496968 | 3608 | 73.09 | NC_000019 | 18496968 |
| MRPL32 | **-1.44** | **0.01** | 1576 | 62.91 | NC_000007 | 42971804 | 2535 | 43.66 | NC_000007 | 42971804 |
| GARS | **-1.44** | **0.01** | 3959 | 60.02 | NC_000007 | 30634181 | 5283 | 41.54 | NC_000007 | 30634181 |
| PSMC1 | **-1.45** | **0.03** | 1547 | 40.73 | NC_000014 | 90722894 | 1977 | 28.17 | NC_000014 | 90722894 |
| FAM50A | **-1.45** | **0.03** | 1127 | 41.01 | NC_000023 | 153672473 | 1473 | 28.34 | NC_000023 | 153672473 |
| PSME2 | **-1.46** | **0.00** | 1829 | 92.44 | NC_000014 | 24612574 | 2453 | 63.19 | NC_000014 | 24612574 |
| IST1 | **-1.46** | **0.01** | 6713 | 68.88 | NC_000016 | 71928311 | 9165 | 47.25 | NC_000016 | 71928311 |
| ITPA | **-1.46** | **0.01** | 2407 | 62.82 | NC_000020 | 3189514 | 4678 | 42.95 | NC_000020 | 3189514 |
| PPIF | **-1.46** | **0.05** | 1574 | 34.42 | NC_000010 | 81107220 | 2016 | 23.62 | NC_000010 | 81107220 |
| PSMD11 | **-1.46** | **0.05** | 3252 | 33.7 | NC_000017 | 30771481 | 4265 | 23.06 | NC_000017 | 30771481 |
| CCNL1 | **-1.47** | **0.00** | 8033 | 74.03 | NC_000003 | 156864291 | 10571 | 50.53 | NC_000003 | 156864291 |
| SRSF3 | **-1.47** | **0.02** | 3152 | 46.99 | NC_000006 | 36562090 | 4402 | 31.94 | NC_000006 | 36562090 |
| PES1 | **-1.47** | **0.04** | 1587 | 34.49 | NC_000022 | 30972612 | 2020 | 23.4 | NC_000022 | 30972612 |
| MRPL27 | **-1.49** | **0.01** | 1118 | 53.31 | NC_000017 | 48445228 | 1485 | 35.67 | NC_000017 | 48445228 |
| ZNF330 | **-1.49** | **0.05** | 1306 | 32.7 | NC_000004 | 142142049 | 1953 | 21.93 | NC_000004 | 142142049 |
| RPL21 | **-1.50** | **0.00** | 4345 | 219.87 | NC_000013 | 27825692 | 5555 | 146.11 | NC_000013 | 27825692 |
| DCXR | **-1.50** | **0.00** | 1203 | 79.24 | NC_000017 | 79993757 | 1511 | 52.83 | NC_000017 | 79993757 |
| PSMC5 | **-1.50** | **0.00** | 2031 | 64.81 | NC_000017 | 61904770 | 2501 | 43.23 | NC_000017 | 61904770 |

| | | | | | | | | | | |
|---|---|---|---|---|---|---|---|---|---|---|
| ATP6V1G1 | **-1.50** | **0.01** | 1933 | 53.43 | NC_000009 | 117349994 | 2267 | 35.52 | NC_000009 | 117349994 |
| GCLC | **-1.50** | **0.03** | 5452 | 39.11 | NC_000006 | 53362139 | 5110 | 26.07 | NC_000006 | 53362139 |
| C9orf142 | **-1.50** | **0.04** | 570 | 33.68 | NC_000009 | 139886870 | 678 | 22.39 | NC_000009 | 139886870 |
| ODC1 | **-1.51** | **0.00** | 6123 | 128.34 | NC_000002 | 10580507 | 7287 | 85.02 | NC_000002 | 10580507 |
| MDM2 | **-1.51** | **0.00** | 13456 | 82.07 | NC_000012 | 69201952 | 15103 | 54.35 | NC_000012 | 69201952 |
| EIF4A3 | **-1.51** | **0.01** | 2058 | 50.83 | NC_000017 | 78109013 | 2282 | 33.77 | NC_000017 | 78109013 |
| BOD1 | **-1.51** | **0.03** | 1670 | 36.11 | NC_000005 | 173034148 | 2723 | 23.84 | NC_000005 | 173034148 |
| RIOK3 | **-1.51** | **0.05** | 2793 | 31.64 | NC_000018 | 21032787 | 3269 | 20.93 | NC_000018 | 21032787 |
| HKDC1 | **-1.51** | **0.05** | 3378 | 29.9 | NC_000010 | 70980059 | 3967 | 19.77 | NC_000010 | 70980059 |
| SF3B2 | **-1.52** | **0.00** | 4582 | 64.22 | NC_000011 | 65819264 | 5488 | 42.38 | NC_000011 | 65819264 |
| OSER1 | **-1.52** | **0.02** | 2397 | 39.66 | NC_000020 | 42824579 | 3594 | 26.15 | NC_000020 | 42824579 |
| EIF1 | **-1.53** | **0.00** | 5881 | 239.54 | NC_000017 | 39845127 | 7249 | 156.97 | NC_000017 | 39845127 |
| MRPL9 | **-1.53** | **0.04** | 936 | 31.76 | NC_000001 | 151732119 | 1176 | 20.75 | NC_000001 | 151732119 |
| HNRNPM | **-1.54** | **0.01** | 4056 | 46.46 | NC_000019 | 8509460 | 4367 | 30.2 | NC_000019 | 8509460 |
| ANO9 | **-1.54** | **0.02** | 2485 | 38.15 | NC_000011 | 417930 | 3241 | 24.83 | NC_000011 | 417930 |
| TNFAIP3 | **-1.54** | **0.02** | 3753 | 37.56 | NC_000006 | 138188325 | 4288 | 24.32 | NC_000006 | 138188325 |
| SRRT | **-1.54** | **0.03** | 2153 | 36.54 | NC_000007 | 100472701 | 2734 | 23.75 | NC_000007 | 100472701 |
| KIAA0020 | **-1.54** | **0.04** | 1642 | 30.78 | NC_000009 | 2804152 | 2309 | 19.93 | NC_000009 | 2804152 |
| YBX3 | **-1.55** | **0.01** | 2757 | 46.73 | NC_000012 | 10851676 | 3978 | 30.18 | NC_000012 | 10851676 |
| PFKFB3 | **-1.55** | **0.04** | 3260 | 30.48 | NC_000010 | 6186843 | 4135 | 19.7 | NC_000010 | 6186843 |
| MYC | **-1.55** | **0.05** | 1177 | 27.16 | NC_000008 | 128748315 | 1512 | 17.47 | NC_000008 | 128748315 |
| CCL20 | **-1.56** | **0.00** | 1574 | 89.85 | NC_000002 | 228678558 | 2098 | 57.76 | NC_000002 | 228678558 |
| KDM3A | **-1.56** | **0.01** | 5766 | 55.06 | NC_000002 | 86668271 | 6785 | 35.24 | NC_000002 | 86668271 |
| EIF2B4 | **-1.57** | **0.01** | 2319 | 53.16 | NC_000002 | 27587219 | 2961 | 33.9 | NC_000002 | 27587219 |
| KLF6 | **-1.57** | **0.02** | 3336 | 39.15 | NC_000010 | 3818188 | 4066 | 24.86 | NC_000010 | 3818188 |
| SIRT7 | **-1.57** | **0.04** | 1303 | 29.92 | NC_000017 | 79869815 | 1770 | 19.1 | NC_000017 | 79869815 |
| HNRNPAB | **-1.57** | **0.04** | 1384 | 29.96 | NC_000005 | 177631508 | 1719 | 19.1 | NC_000005 | 177631508 |
| PSMC4 | **-1.58** | **0.01** | 1901 | 44.55 | NC_000019 | 40476912 | 2248 | 28.16 | NC_000019 | 40476912 |
| SSR4 | **-1.58** | **0.02** | 929 | 39.21 | NC_000023 | 153059630 | 1146 | 24.74 | NC_000023 | 153059630 |
| EFNA1 | **-1.59** | **0.00** | 9036 | 197.77 | NC_000001 | 155100349 | 12193 | 124.41 | NC_000001 | 155100349 |
| GPX1 | **-1.59** | **0.01** | 899 | 43.75 | NC_000003 | 49394609 | 1078 | 27.56 | NC_000003 | 49394609 |
| AURKAIP1 | **-1.59** | **0.04** | 603 | 29.56 | NC_000001 | 1309110 | 713 | 18.62 | NC_000001 | 1309110 |
| RHOB | **-1.60** | **0.00** | 3117 | 75.82 | NC_000002 | 20646835 | 3713 | 47.44 | NC_000002 | 20646835 |
| HNRNPH3 | **-1.60** | **0.00** | 3295 | 61.72 | NC_000010 | 70091768 | 4099 | 38.64 | NC_000010 | 70091768 |
| TNFRSF12A | **-1.61** | **0.01** | 929 | 47.21 | NC_000016 | 3070313 | 1120 | 29.34 | NC_000016 | 3070313 |
| HNRNPU | **-1.61** | **0.01** | 5522 | 42.86 | NC_000001 | 245013602 | 6619 | 2.67E+01 | NC_000001 | 245013602 |
| EWSR1 | **-1.62** | **0.00** | 6488 | 87.96 | NC_000022 | 29663998 | 8894 | 54.32 | NC_000022 | 29663998 |
| TXNRD1 | **-1.62** | **0.03** | 4847 | 29.46 | NC_000012 | 104609537 | 5069 | 18.15 | NC_000012 | 104609537 |
| TIMM17A | **-1.62** | **0.04** | 1056 | 28.18 | NC_000001 | 201924619 | 1370 | 17.37 | NC_000001 | 201924619 |
| HSP90AA1 | **-1.63** | **0.00** | 11039 | 148.53 | NC_000014 | 102547075 | 12834 | 91.08 | NC_000014 | 102547075 |
| SLC25A39 | **-1.63** | **0.00** | 2957 | 69.13 | NC_000017 | 42396993 | 3295 | 42.37 | NC_000017 | 42396993 |
| TFRC | **-1.63** | **0.00** | 6653 | 64.54 | NC_000003 | 195776155 | 7556 | 39.49 | NC_000003 | 195776155 |
| C17orf89 | **-1.63** | **0.02** | 617 | 35.85 | NC_000017 | 79213039 | 795 | 22.02 | NC_000017 | 79213039 |
| UCKL1 | **-1.63** | **0.04** | 1330 | 26.56 | NC_000020 | 62571182 | 1831 | 16.3 | NC_000020 | 62571182 |
| CXorf40B | **-1.63** | **0.05** | 1286 | 23.67 | NC_000023 | 149100415 | 1743 | 14.53 | NC_000023 | 149100415 |
| NGDN | **-1.63** | **0.05** | 774 | 24.33 | NC_000014 | 23938898 | 927 | 14.89 | NC_000014 | 23938898 |
| RPL13 | **-1.64** | **0.00** | 6538 | 57.82 | NC_000016 | 89613324 | 7508 | 35.32 | NC_000016 | 89613324 |
| CIR1 | **-1.64** | **0.02** | 1355 | 35.1 | NC_000002 | 175212878 | 1744 | 21.34 | NC_000002 | 175212878 |
| BRIX1 | **-1.64** | **0.04** | 745 | 25.28 | NC_000005 | 34915820 | 799 | 15.37 | NC_000005 | 34915820 |
| EEF1D | **-1.65** | **0.00** | 3684 | 52.23 | NC_000008 | 144661867 | 4900 | 31.59 | NC_000008 | 144661867 |
| SARS | **-1.65** | **0.01** | 2323 | 39.16 | NC_000001 | 109756515 | 2394 | 23.79 | NC_000001 | 109756515 |
| RRP1 | **-1.65** | **0.03** | 1659 | 28.04 | NC_000021 | 45209418 | 1769 | 16.95 | NC_000021 | 45209418 |
| MKI67IP | **-1.65** | **0.05** | 1011 | 23.09 | NC_000002 | 122484521 | 1240 | 13.99 | NC_000002 | 122484521 |
| DNAJB11 | **-1.66** | **0.00** | 3311 | 81.5 | NC_000003 | 186288467 | 4390 | 49.21 | NC_000003 | 186288467 |
| DDX47 | **-1.66** | **0.01** | 1875 | 46.13 | NC_000012 | 12966280 | 2239 | 27.78 | NC_000012 | 12966280 |
| CLDN4 | **-1.66** | **0.01** | 1268 | 40.05 | NC_000007 | 73245193 | 1454 | 24.12 | NC_000007 | 73245193 |
| YKT6 | **-1.67** | **0.01** | 2470 | 40.17 | NC_000007 | 44240577 | 2840 | 24 | NC_000007 | 44240577 |
| UAP1L1 | **-1.67** | **0.05** | 1601 | 23.98 | NC_000009 | 139971944 | 1873 | 14.37 | NC_000009 | 139971944 |
| TRAF4 | **-1.68** | **0.00** | 3083 | 53.47 | NC_000017 | 27071023 | 3770 | 31.86 | NC_000017 | 27071023 |
| ELF3 | **-1.69** | **0.01** | 2910 | 42.05 | NC_000001 | 201979647 | 3247 | 24.94 | NC_000001 | 201979647 |
| C19orf80 | **-1.70** | **0.00** | 1483 | 85.5 | NC_000019 | 11350295 | 1726 | 50.36 | NC_000019 | 11350295 |
| ATP6V1B2 | **-1.72** | **0.04** | 1471 | 23.77 | NC_000008 | 20054704 | 1674 | 13.84 | NC_000008 | 20054704 |
| ZNF207 | **-1.73** | **0.00** | 2745 | 44.11 | NC_000017 | 30677071 | 2936 | 25.52 | NC_000017 | 30677071 |
| RBPJ | **-1.73** | **0.01** | 4905 | 35.83 | NC_000004 | 26165077 | 4912 | 20.68 | NC_000004 | 26165077 |
| ETF1 | **-1.74** | **0.03** | 2399 | 25.33 | NC_000005 | 137841782 | 2535 | 14.58 | NC_000005 | 137841782 |
| EIF3G | **-1.75** | **0.00** | 1989 | 64.83 | NC_000019 | 10225690 | 2040 | 37.06 | NC_000019 | 10225690 |
| ZFP36 | **-1.76** | **0.00** | 3041 | 95.89 | NC_000019 | 39897487 | 3381 | 54.58 | NC_000019 | 39897487 |
| DNPEP | **-1.76** | **0.01** | 2316 | 32.4 | NC_000002 | 220238180 | 2529 | 18.38 | NC_000002 | 220238180 |

| | | | | | | | | | | |
|---|---|---|---|---|---|---|---|---|---|---|
| AARSD1 | **-1.76** | **0.03** | 1011 | 24.65 | NC_000017 | 41102543 | 1108 | 13.97 | NC_000017 | 41102543 |
| RIT1 | **-1.76** | **0.04** | 1600 | 22 | NC_000001 | 155867599 | 1624 | 12.47 | NC_000001 | 155867599 |
| PPP1R15A | **-1.77** | **0.00** | 3023 | 62.65 | NC_000019 | 49375649 | 3036 | 35.4 | NC_000019 | 49375649 |
| UGDH | **-1.77** | **0.00** | 5551 | 53.2 | NC_000004 | 39500375 | 4396 | 30 | NC_000004 | 39500375 |
| ZNRD1 | **-1.77** | **0.02** | 583 | 28.52 | NC_000006 | 30029017 | 579 | 16.12 | NC_000006 | 30029017 |
| CCDC107 | **-1.77** | **0.03** | 743 | 26.1 | NC_000009 | 35658279 | 975 | 14.74 | NC_000009 | 35658279 |
| CLK1 | **-1.78** | **0.00** | 7968 | 154.61 | NC_000002 | 201717732 | 9187 | 86.96 | NC_000002 | 201717732 |
| GNL2 | **-1.78** | **0.00** | 2570 | 48.03 | NC_000001 | 38032413 | 2842 | 27.02 | NC_000001 | 38032413 |
| NT5C | **-1.78** | **0.01** | 691 | 38.62 | NC_000017 | 73126320 | 743 | 21.71 | NC_000017 | 73126320 |
| GTPBP4 | **-1.78** | **0.01** | 2121 | 35.27 | NC_000010 | 1034349 | 2217 | 19.79 | NC_000010 | 1034349 |
| ARL8B | **-1.79** | **0.02** | 3211 | 29.95 | NC_000003 | 5163930 | 2122 | 16.73 | NC_000003 | 5163930 |
| IFRD1 | **-1.81** | **0.01** | 3068 | 33.44 | NC_000007 | 112063199 | 4148 | 18.47 | NC_000007 | 112063199 |
| PSME4 | **-1.81** | **0.01** | 5280 | 29.56 | NC_000002 | 54091204 | 5356 | 16.36 | NC_000002 | 54091204 |
| GRB10 | **-1.81** | **0.01** | 7454 | 31.25 | NC_000007 | 50657760 | 7836 | 17.27 | NC_000007 | 50657760 |
| DNAJB1 | **-1.82** | **0.00** | 2264 | 48.04 | NC_000019 | 14625581 | 2398 | 26.44 | NC_000019 | 14625581 |
| MAP2K3 | **-1.82** | **0.01** | 1873 | 29.09 | NC_000017 | 21187968 | 2114 | 15.95 | NC_000017 | 21187968 |
| SUPV3L1 | **-1.83** | **0.02** | 1666 | 28.21 | NC_000010 | 70939993 | 1974 | 15.46 | NC_000010 | 70939993 |
| SAT1 | **-1.84** | **0.00** | 28282 | 1,085.02 | NC_000023 | 23801275 | 37210 | 590.7 | NC_000023 | 23801275 |
| WDR45B | **-1.84** | **0.00** | 6189 | 87.36 | NC_000017 | 80572438 | 6108 | 47.47 | NC_000017 | 80572438 |
| DNAJC2 | **-1.84** | **0.00** | 1976 | 43.94 | NC_000007 | 102952921 | 2150 | 23.94 | NC_000007 | 102952921 |
| SRSF6 | **-1.85** | **0.00** | 12521 | 177.79 | NC_000020 | 42086504 | 13255 | 96.27 | NC_000020 | 42086504 |
| RSL1D1 | **-1.85** | **0.04** | 2082 | 19.96 | NC_000016 | 11928053 | 2135 | 10.81 | NC_000016 | 11928053 |
| CDKN1A | **-1.86** | **0.01** | 2002 | 36 | NC_000006 | 36644237 | 2032 | 19.39 | NC_000006 | 36644237 |
| RP9 | **-1.86** | **0.01** | 783 | 30.51 | NC_000007 | 33134409 | 1044 | 16.4 | NC_000007 | 33134409 |
| THOC6 | **-1.86** | **0.02** | 674 | 24.77 | NC_000016 | 3074032 | 669 | 13.31 | NC_000016 | 3074032 |
| RRN3 | **-1.86** | **0.04** | 1625 | 20.66 | NC_000016 | 15153879 | 1666 | 11.11 | NC_000016 | 15153879 |
| SFN | **-1.87** | **0.01** | 823 | 36.03 | NC_000001 | 27189633 | 837 | 19.25 | NC_000001 | 27189633 |
| SMAD7 | **-1.87** | **0.02** | 1932 | 26.05 | NC_000018 | 46446223 | 1860 | 13.93 | NC_000018 | 46446223 |
| RPS17 | **-1.88** | **0.00** | 949 | 84.88 | NC_000015 | 82821161 | 821 | 45.15 | NC_000015 | 82821161 |
| PFDN2 | **-1.88** | **0.00** | 794 | 49.86 | NC_000001 | 161070346 | 723 | 26.52 | NC_000001 | 161070346 |
| GUK1 | **-1.88** | **0.01** | 1707 | 35.1 | NC_000001 | 228327668 | 2064 | 18.72 | NC_000001 | 228327668 |
| ZFAND5 | **-1.88** | **0.02** | 3451 | 25.79 | NC_000009 | 74966341 | 3690 | 13.68 | NC_000009 | 74966341 |
| RSL24D1 | **-1.89** | **0.00** | 1503 | 50.58 | NC_000015 | 55473512 | 1587 | 26.75 | NC_000015 | 55473512 |
| MSMO1 | **-1.89** | **0.01** | 1814 | 28.05 | NC_000004 | 166248818 | 1809 | 14.81 | NC_000004 | 166248818 |
| FAM102A | **-1.89** | **0.05** | 1588 | 17.55 | NC_000009 | 130702861 | 1497 | 9.29 | NC_000009 | 130702861 |
| ZFAND2A | **-1.90** | **0.00** | 3468 | 128.76 | NC_000007 | 1192543 | 3513 | 67.67 | NC_000007 | 1192543 |
| DUSP1 | **-1.92** | **0.00** | 2734 | 72.71 | NC_000005 | 172195093 | 2737 | 37.95 | NC_000005 | 172195093 |
| EIF3A | **-1.93** | **0.00** | 6241 | 41.61 | NC_000010 | 120794541 | 5157 | 2.15E+01 | NC_000010 | 120794541 |
| FOSL1 | **-1.93** | **0.01** | 3924 | 33.21 | NC_000011 | 65659616 | 1595 | 17.19 | NC_000011 | 65659616 |
| ISCU | **-1.93** | **0.01** | 2006 | 28.29 | NC_000012 | 108955239 | 2240 | 14.68 | NC_000012 | 108955239 |
| TRIB1 | **-1.93** | **0.04** | 1440 | 18.77 | NC_000008 | 126442563 | 1345 | 9.72 | NC_000008 | 126442563 |
| HGFAC | **-1.94** | **0.03** | 962 | 20.06 | NC_000004 | 3443702 | 1052 | 10.33 | NC_000004 | 3443702 |
| ARFGAP1 | **-1.96** | **0.00** | 12016 | 178.21 | NC_000020 | 61904137 | 13385 | 91.02 | NC_000020 | 61904137 |
| UBXN1 | **-1.96** | **0.00** | 1393 | 49.48 | NC_000011 | 62443972 | 1304 | 25.19 | NC_000011 | 62443972 |
| HRAS | **-1.96** | **0.04** | 412 | 18.13 | NC_000011 | 532242 | 399 | 9.23 | NC_000011 | 532242 |
| HGS | **-1.97** | **0.00** | 2172 | 36.7 | NC_000017 | 79650962 | 2276 | 18.62 | NC_000017 | 79650962 |
| PPP1R15B | **-1.97** | **0.02** | 2184 | 22.55 | NC_000001 | 204369781 | 2119 | 11.46 | NC_000001 | 204369781 |
| GPBP1 | **-1.98** | **0.01** | 3988 | 32.13 | NC_000005 | 56469676 | 3832 | 16.24 | NC_000005 | 56469676 |
| NCOR1 | **-1.98** | **0.05** | 4390 | 15.4 | NC_000017 | 15933408 | 4062 | 7.79 | NC_000017 | 15933408 |
| KLF10 | **-1.99** | **0.02** | 1621 | 23.58 | NC_000008 | 103661005 | 1484 | 11.85 | NC_000008 | 103661005 |
| NFIL3 | **-2.01** | **0.00** | 2020 | 45.17 | NC_000009 | 94171327 | 1990 | 22.48 | NC_000009 | 94171327 |
| RBMX2 | **-2.01** | **0.02** | 650 | 20.72 | NC_000023 | 129535943 | 840 | 10.29 | NC_000023 | 129535943 |
| WDR74 | **-2.04** | **0.00** | 1910 | 33.41 | NC_000011 | 62600380 | 1833 | 16.35 | NC_000011 | 62600380 |
| NXF1 | **-2.04** | **0.03** | 1507 | 17.53 | NC_000011 | 62559597 | 1338 | 8.58 | NC_000011 | 62559597 |
| SQSTM1 | **-2.06** | **0.00** | 12647 | 163.89 | NC_000005 | 179233388 | 11513 | 79.53 | NC_000005 | 179233388 |
| RRP9 | **-2.07** | **0.03** | 600 | 17.54 | NC_000003 | 51967442 | 522 | 8.47 | NC_000003 | 51967442 |
| BLOC1S2 | **-2.08** | **0.02** | 1314 | 19.87 | NC_000010 | 102033035 | 1193 | 9.53 | NC_000010 | 102033035 |
| HSPA1B | **-2.11** | **0.00** | 5062 | 91.92 | NC_000006 | 31789964 | 4569 | 43.55 | NC_000006 | 31789964 |
| ETS2 | **-2.12** | **0.00** | 8294 | 65.58 | NC_000021 | 40177231 | 6797 | 30.88 | NC_000021 | 40177231 |
| WTAP | **-2.12** | **0.00** | 4395 | 48.45 | NC_000006 | 160147712 | 4105 | 22.87 | NC_000006 | 160147712 |
| POLR1C | **-2.14** | **0.00** | 1889 | 39.94 | NC_000006 | 43484777 | 2141 | 18.65 | NC_000006 | 43484777 |
| PIM3 | **-2.14** | **0.00** | 1503 | 35.87 | NC_000022 | 50354143 | 1328 | 16.73 | NC_000022 | 50354143 |
| MIF | **-2.16** | **0.01** | 226 | 23.09 | NC_000022 | 24236565 | 198 | 10.67 | NC_000022 | 24236565 |
| PTP4A1 | **-2.17** | **0.00** | 9351 | 92.01 | NC_000006 | 64281917 | 8464 | 42.48 | NC_000006 | 64281917 |
| PAK1IP1 | **-2.18** | **0.01** | 930 | 20.94 | NC_000006 | 10695180 | 650 | 9.61 | NC_000006 | 10695180 |
| BIRC7 | **-2.19** | **0.03** | 576 | 16.29 | NC_000020 | 61867235 | 643 | 7.43 | NC_000020 | 61867235 |
| UTP6 | **-2.20** | **0.00** | 4380 | 80.32 | NC_000017 | 30190190 | 4238 | 36.56 | NC_000017 | 30190190 |
| EIF5B | **-2.20** | **0.00** | 4587 | 51.6 | NC_000002 | 99953821 | 4002 | 23.5 | NC_000002 | 99953821 |

| | | | | | | | | | | |
|---|---|---|---|---|---|---|---|---|---|---|
| TRIB3 | **-2.23** | **0.00** | 3049 | 52.57 | NC_000020 | 356447 | 2762 | 23.62 | NC_000020 | 356447 |
| ABCF1 | **-2.23** | **0.00** | 1982 | 29.28 | NC_000006 | 30539170 | 1633 | 13.16 | NC_000006 | 30539170 |
| MSANTD3 | **-2.23** | **0.02** | 1511 | 18.22 | NC_000009 | 103189495 | 1454 | 8.15 | NC_000009 | 103189495 |
| CDK9 | **-2.23** | **0.05** | 582 | 12.6 | NC_000009 | 130548305 | 526 | 5.65 | NC_000009 | 130548305 |
| FDXR | **-2.25** | **0.00** | 2032 | 33.5 | NC_000017 | 72858619 | 1770 | 14.9 | NC_000017 | 72858619 |
| ALYREF | **-2.29** | **0.00** | 1481 | 52.04 | NC_000017 | 79845711 | 1202 | 22.69 | NC_000017 | 79845711 |
| LPAR2 | **-2.29** | **0.03** | 485 | 15.19 | NC_000019 | 19734464 | 477 | 6.63 | NC_000019 | 19734464 |
| PIM1 | **-2.31** | **0.00** | 1474 | 29.12 | NC_000006 | 37137922 | 1189 | 12.63 | NC_000006 | 37137922 |
| JUN | **-2.32** | **0.00** | 2066 | 35.8 | NC_000001 | 59246463 | 1697 | 15.45 | NC_000001 | 59246463 |
| DDX39A | **-2.35** | **0.00** | 2201 | 69.35 | NC_000019 | 14519610 | 1869 | 29.52 | NC_000019 | 14519610 |
| JUNB | **-2.36** | **0.05** | 377 | 11.95 | NC_000019 | 12902310 | 304 | 5.06 | NC_000019 | 12902310 |
| ZNF593 | **-2.37** | **0.01** | 285 | 23.24 | NC_000001 | 26496388 | 220 | 9.79 | NC_000001 | 26496388 |
| WDR43 | **-2.38** | **0.00** | 5537 | 63.4 | NC_000002 | 29117533 | 4707 | 26.67 | NC_000002 | 29117533 |
| PDGFA | **-2.43** | **0.04** | 1073 | 13.06 | NC_000007 | 536895 | 863 | 5.38 | NC_000007 | 536895 |
| RPS28 | **-2.46** | **0.00** | 841 | 107.16 | NC_000019 | 8386384 | 650 | 43.55 | NC_000019 | 8386384 |
| COL7A1 | **-2.46** | **0.00** | 4452 | 25.72 | NC_000003 | 48601506 | 3498 | 10.45 | NC_000003 | 48601506 |
| RRP12 | **-2.50** | **0.02** | 1702 | 14.74 | NC_000010 | 99116458 | 1223 | 5.9 | NC_000010 | 99116458 |
| ID2 | **-2.51** | **0.00** | 37225 | 1,154.13 | NC_000002 | 8822113 | 27209 | 460.21 | NC_000002 | 8822113 |
| NFKBIA | **-2.54** | **0.00** | 4362 | 149.17 | NC_000014 | 35870716 | 3266 | 58.73 | NC_000014 | 35870716 |
| ISG15 | **-2.55** | **0.00** | 347 | 29.26 | NC_000001 | 948847 | 265 | 11.47 | NC_000001 | 948847 |
| EGR1 | **-2.56** | **0.01** | 921 | 16.91 | NC_000005 | 137801181 | 688 | 6.62 | NC_000005 | 137801181 |
| DDIT3 | **-2.58** | **0.00** | 3843 | 147.4 | NC_000012 | 57910371 | 2942 | 57.23 | NC_000012 | 57910371 |
| MRPS30 | **-2.60** | **0.00** | 982 | 25.62 | NC_000005 | 44809027 | 712 | 9.85 | NC_000005 | 44809027 |
| STK17A | **-2.66** | **0.03** | 1474 | 12.52 | NC_000007 | 43622692 | 966 | 4.71 | NC_000007 | 43622692 |
| TGIF2-C20orf24 | **-2.69** | **0.00** | 2689 | 78.07 | NC_000020 | 35202957 | 2998 | 28.97 | NC_000020 | 35202957 |
| RPF2 | **-2.70** | **0.00** | 959 | 22.11 | NC_000006 | 111303274 | 679 | 8.2 | NC_000006 | 111303274 |
| GADD45A | **-2.77** | **0.00** | 1295 | 46.39 | NC_000001 | 68150860 | 929 | 16.74 | NC_000001 | 68150860 |
| UPP1 | **-2.83** | **0.00** | 3428 | 42.24 | NC_000007 | 48128355 | 3703 | 14.95 | NC_000007 | 48128355 |
| ULK1 | **-3.03** | **0.01** | 1515 | 14.58 | NC_000012 | 132379279 | 958 | 4.81 | NC_000012 | 132379279 |
| HSPH1 | **-3.04** | **0.00** | 2626 | 27.54 | NC_000013 | 31710762 | 1913 | 9.07 | NC_000013 | 31710762 |
| PBDC1 | **-3.07** | **0.00** | 791 | 31.03 | NC_000023 | 75392771 | 490 | 10.1 | NC_000023 | 75392771 |
| SAT2 | **-3.08** | **0.00** | 4780 | 245 | NC_000017 | 7529552 | 2877 | 79.57 | NC_000017 | 7529552 |
| PVRL4 | **-3.09** | **0.02** | 735 | 11.66 | NC_000001 | 161040781 | 458 | 3.78 | NC_000001 | 161040781 |
| JUND | **-3.14** | **0.00** | 2080 | 64.04 | NC_000019 | 18390563 | 1262 | 20.41 | NC_000019 | 18390563 |
| SLC20A1 | **-3.21** | **0.00** | 11908 | 162.2 | NC_000002 | 113403434 | 7266 | 50.61 | NC_000002 | 113403434 |
| RND1 | **-3.35** | **0.00** | 1013 | 18.81 | NC_000012 | 49250916 | 476 | 5.61 | NC_000012 | 49250916 |
| RRAD | **-3.37** | **0.00** | 1038 | 21.1 | NC_000016 | 66955582 | 507 | 6.27 | NC_000016 | 66955582 |
| GADD45B | **-3.45** | **0.00** | 1969 | 77.34 | NC_000019 | 2476123 | 1080 | 22.41 | NC_000019 | 2476123 |
| PDIA2 | **-3.46** | **0.03** | 303 | 9.37 | NC_000016 | 333118 | 173 | 2.71 | NC_000016 | 333118 |
| SESN2 | **-3.53** | **0.00** | 1287 | 16.42 | NC_000001 | 28585963 | 647 | 4.65 | NC_000001 | 28585963 |
| SIK1 | **-3.67** | **0.00** | 3877 | 43.14 | NC_000021 | 44834395 | 1985 | 11.77 | NC_000021 | 44834395 |
| UBALD1 | **-3.69** | **0.05** | 335 | 7.42 | NC_000016 | 4658884 | 326 | 2.01 | NC_000016 | 4658884 |
| CWF19L2 | **-3.74** | **0.04** | 614 | 7.55 | NC_000011 | 107197071 | 417 | 2.02 | NC_000011 | 107197071 |
| NUPR1 | **-3.81** | **0.00** | 597 | 27.4 | NC_000016 | 28548662 | 267 | 7.2 | NC_000016 | 28548662 |
| MTRNR2L9 | **-3.81** | **0.05** | 62 | 6.77 | NC_000006 | 62284008 | 31 | 1.78 | NC_000006 | 62284008 |
| ATF3 | **-3.92** | **0.00** | 1133 | 18.81 | NC_000001 | 212738676 | 622 | 4.8 | NC_000001 | 212738676 |
| CEBPB | **-4.08** | **0.00** | 583 | 15.92 | NC_000020 | 48807120 | 272 | 3.9 | NC_000020 | 48807120 |
| PLK3 | **-4.48** | **0.00** | 2271 | 42.51 | NC_000001 | 45265638 | 882 | 9.48 | NC_000001 | 45265638 |
| PLK2 | **-4.64** | **0.00** | 812 | 15.55 | NC_000005 | 57749809 | 354 | 3.36 | NC_000005 | 57749809 |
| HBA1 | **-41.88** | **0.00** | 88 | 8.8 | NC_000016 | 226679 | 4 | 0.21 | NC_000016 | 226679 |
| HBB | **-57.74** | **0.00** | 91 | 8.37 | NC_000011 | 5246696 | 3 | 0.14 | NC_000011 | 5246696 |

**Table S2:** List of all the host transcripts and fold changes that occurred due to *Pb*MLFK-KO infection compared with WT parasite infection in HepG2 cells.

**Table S3**

**List of primers used in this study**

| S.NO | Primer name | Sequence |
|---|---|---|
| 1 | IDL1-FP | 5'CGGGGTACCATGGACTACAAAGATGACGACGAT3' |
| 2 | IDL2-RP | 5'ATTCTCGAGCTCGAGCAGTTTGCAGCTCCACC3' |
| 3 | IDL3-FP | 5'CTCTGCTGGCCCTGGCGCATTCTGCGCACAAC3' |
| 4 | IDL4-RP | 5'GTTGTGCGCAGAATGCGCCAGGGCCAGCAGAG3' |
| 5 | IDL5-FP | 5'GCTCCGAAACTGCACGCAATAGCATATGCAGTTGACAACACC3' |
| 6 | IDL6-RP | 5'GGTGTTGTCAACTGCATATGCTATTGCGTGCAGTTTCGGAGC3' |
| 7 | IDL7-FP | 5'ATATCTCGAGCATAAGGGAAAAGTATACATAATTCA3' |
| 8 | IDL8-RP | 5'CCATCGATGCGTTTGTATGCATTGTTATTCAT 3' |
| 9 | IDL9-FP | 5'ATAAGAATGCGGCCGCGTGGTTGTATTTTTGCG AATGTAT3' |
| 10 | IDL10-RP | 5'TCCCCGCGGTTGTT GTATAGGATTCCTTCTTGA3' |
| *11 | FP-A2M | 5'CAACAACCGCCTGTTACTGC3' |
| 12 | RP-A2M | 5'CATGCTGTATTCCCCAGGCA3' |
| 13 | FP-IL18 | 5'GACCAAGGAAATCGGCCTCT3' |
| 14 | RP-IL18 | 5'TGGTCCGGGGTGCATTATCT3' |
| 15 | FP-HBB | 5'AGAAGTCTGCCGTTACTGC3' |
| 16 | RP-HBB | 5'CAAGGGTAGACCACCAGCA3' |
| 17 | FP- COL5A2 | 5'GCACGCTTGCCCATCATAGA3' |
| 18 | RP- COL5A2 | 5'CCAATTTCAACGCCGAATTC3' |
| 19 | FP-APOH | 5'TGGCCCATCAACACTCTGA3' |
| 20 | RP-APOH | 5'GTCGTATAGCGTACGGCTC3' |
| 21 | FP- B2M | 5'GGAGGCTATCCAGCGTACTC3' |
| 22 | RP- B2M | 5'TGGATGAAACCCAGACACAT3' |
| 23 | FP- HSP90AB1 | 5'ACTCAGCTTTTGTGGAG3' |
| 24 | RP- HSP90AB1 | 5'AATGGGCTCGGTCATATA3' |
| 25 | FP- RPLP0 | 5'ACTCTGCATTCTCGCTTC3' |
| 26 | RP- RPLP0 | 5'ACTCGTTTGTACCCGTTGA3' |
| 27 | FP- TGFB3 | 5'ATGACCCACGTCCCCTATCA3' |
| 28 | RP- TGFB3 | 5'TCCGACTCGGTGTTTTCCTG3' |
| 29 | FP- PRKCB | 5'TGGATTGGTGGGCATTTGGA3' |
| 30 | RP- PRKCB | 5'CAGATGGCCACAGCTTCCTT3' |
| 31 | FP- CASP8 | 5'CAGAGCCTGAGAGAGCGATGT3' |
| 32 | RP- CASP8 | 5'AGTAGGCTGAGGCATCTGTTTC3' |
| 33 | FP- RPL26L1 | 5'GCATTCACCCAAGCAAGGTG3' |
| 34 | RP- RPL26L1 | 5'TCGAGACTTGGCTTTGCGTT3' |

*** S.NO 11 to S.NO 34 primers used for NGS transcriptomic data validation by real time PCR**

**Table S3:** List showing primers, along with their sequence and identities, which were used in this study.

**3. Supplementary Figures:**

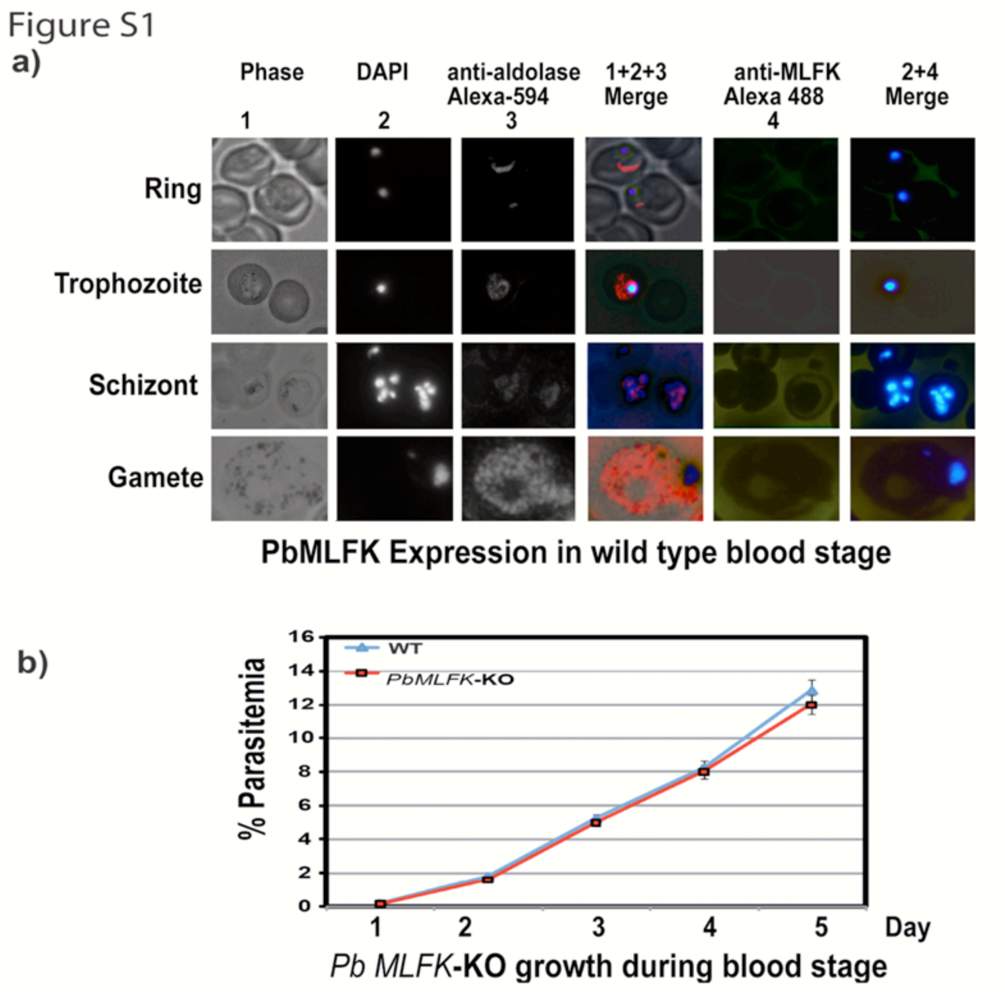


**Figure S1: (A)** Expression of *Pb*MLFK during blood stages of *Pb*A parasites. There was no detectable protein expression in blood stages even at 10 times higher antibody concentration (1:100 dilution for blood stage vs 1:1000 for sporozoite) used in IFA. The nucleus of the parasite is stained with DAPI (blue) and parasite cytoplasm is stained with anti-*Plasmodium* Aldolase Rabbit polyclonal (red). Merged (1+2+3) image shows the overlay Phase, DAPI and Aldolase images. MLFK was stained with anti-MLFK mouse polyclonal antibody (green). Merge (2+4) shows overlay of MLFK and DAPI staining. (B) Growth during the blood stage: There is no significant difference in growth of *PbMLFK*-KO and WT parasites during the blood stage. In this experiment, one million *PbMLFK*-KO or WT parasites were injected intravenously in C57BL/6 mice. Each data point shows the average parasitemia, on that day, from the five mice, along with standard deviation.

## Figure S2

**a)** ***Pb*MLFK N-terminus (1-60 amino acid residues) aligned with signal peptide sequence from ATIA2_RAT (Eukaryote)**

```
PbMLFK  1 MIDSADKIEYSKEKTLACNGGNKDYEQDESSLKYKKTLDMDTIENDFYNCMEKESICDEK 60
                  EYS   T A NGG K  ++++   + KK + MD
AT1A2_RAT       8 EYSPAATTAENGGGKKKQKEKELDELKKEVAMD 40

Identities = 13/33 (39%), Positives = 19/33 (57%)
```

**b)** ***Pb*MLFK N-terminus (1-60 amino acid residues) aligned with signal peptide sequence from SEPF_CLOCE (Prokaryote)**

```
PbMLFK 1  MIDSADKIEYSKEKTLACNGGNKDYEQDESSLKYKKTLDMDTIENDFYNCMEKESICDEK 60
                                 +YE DE  L  K+ +  + I+  F+N  +K+
SEPF_CLOCE                    21 EYEYDEQELNTKEEVKDEPIQTHFFNGSKKQ 51
```

Identities = 10/31 (32%), Positives = 18/31 (58%)

**c)** **Transmembrane region prediction using TMpred bioinformatics tool**

Prediction of transmembrane domain

The sequence positions in brackets denominate the core region.
Only scores above 500 are considered significant.
Inside to outside helices : 1 found

| from | to | score | center |
|---|---|---|---|
| 1111 (1111) | 1130 (1128) | 1554 | 1120 |

Outside to inside helices : 1 found

| from | to | score | center |
|---|---|---|---|
| 1111 (1111) | 1127 (1127) | 1003 | 1119 |

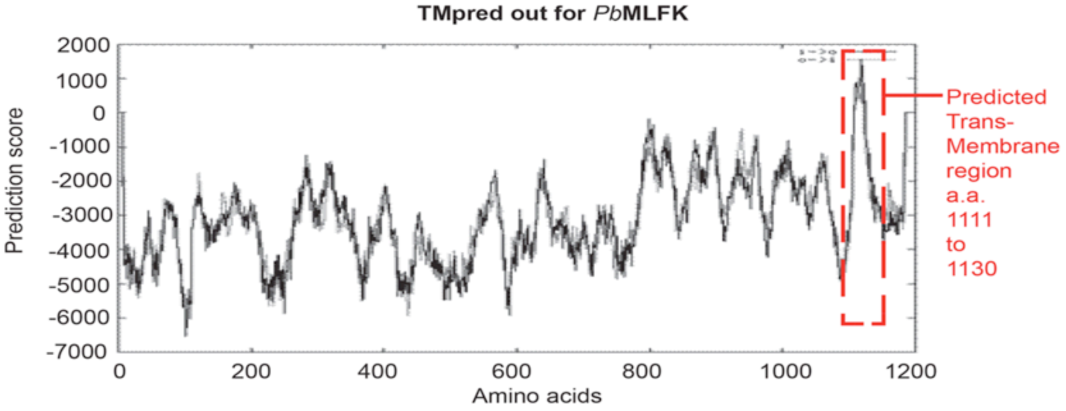


**Figure S2: *Pb*MLFK N-terminus BLAST search against known signal peptides and prediction of the trans-membrane region.** (A, B) *Pb*MLFK (amino acids 1-60 from the N-terminus) aligned with signal peptide sequence from ATIA2_RAT (A) and SEPF_CLOCE (B), which were the best matches. Both A and B together highlight the absence of a typical signal sequence. (C) Prediction of the trans-membrane region in *Pb*MLFK using TMpred. The TMpred program predicts the presence of a trans-membrane domain with high confidence in the region of amino acids 1111-1130.

**Full blots figure 1d**

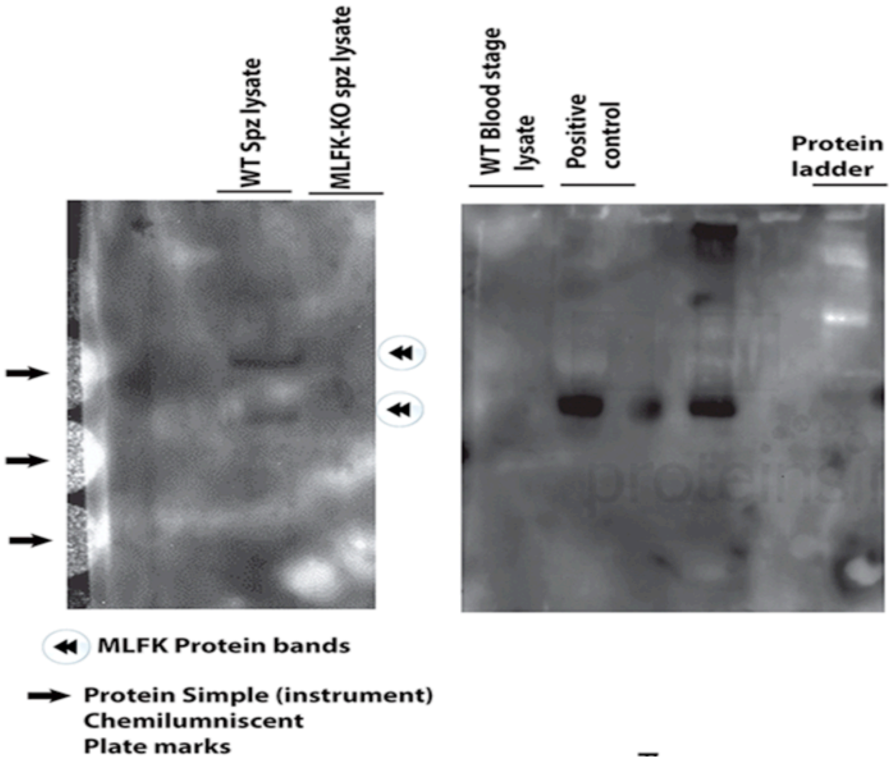


**Full blot figure 1e**

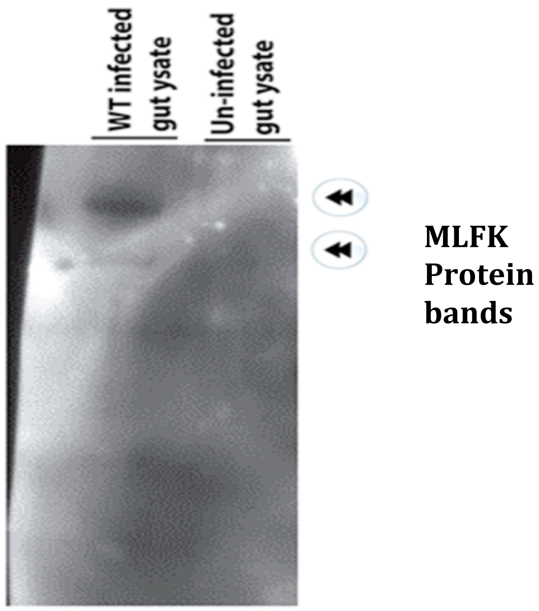

**Full image figure 2 a**

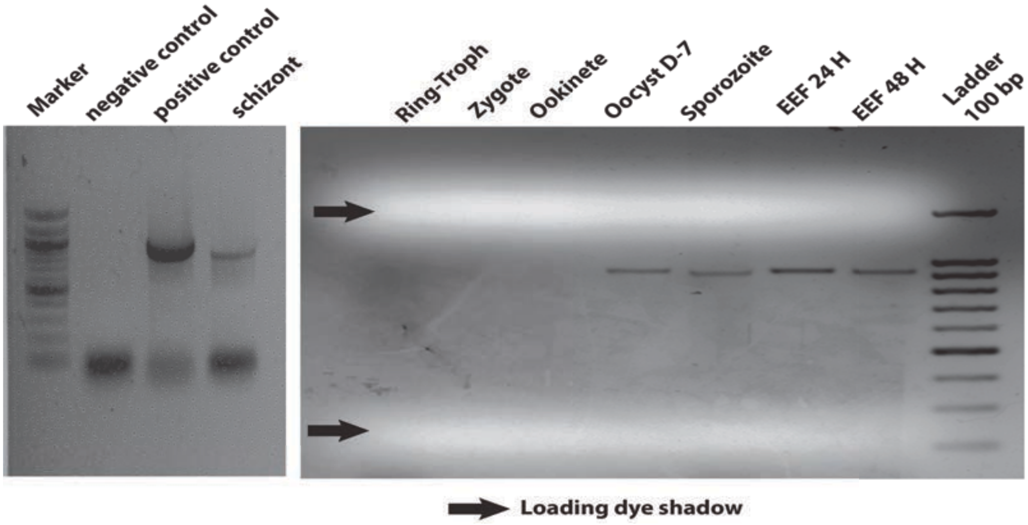


**Full image figure 3 c**

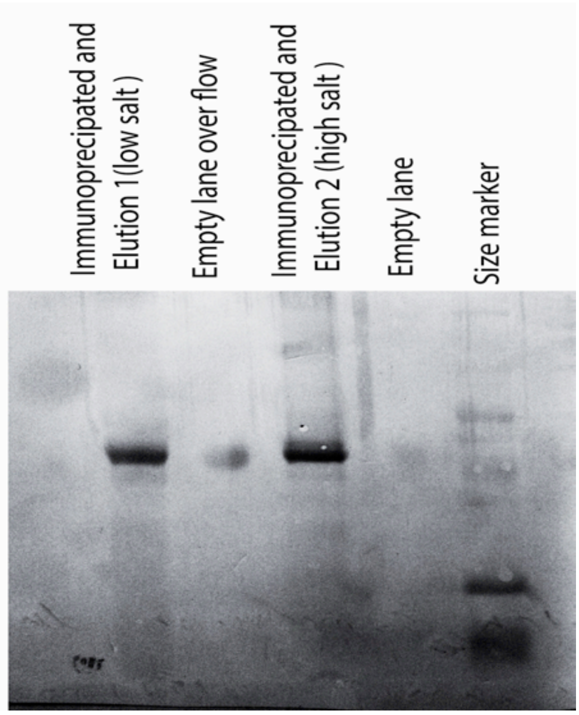

**Full blot figure 4 b**

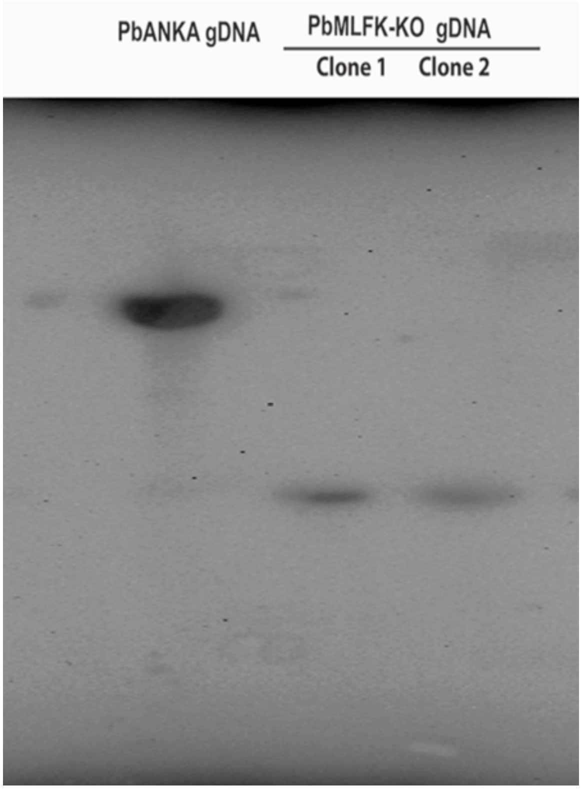

Supplement: Supplementary Information [file srep39285-s1.pdf]
